# Inhibition of VMAT2 by β2-adrenergic agonists, antagonists, and the atypical antipsychotic ziprasidone

**DOI:** 10.1038/s42003-022-04121-1

**Published:** 2022-11-23

**Authors:** Svein Isungset Støve, Åge Aleksander Skjevik, Knut Teigen, Aurora Martinez

**Affiliations:** 1 Department of Biomedicine, University of Bergen, Jonas Lies vei 91, 5009 Bergen, Norway; 2 Neuro-SysMed, Department of Neurology, Haukeland University Hospital, 5021 Bergen, Norway; 3 K.G. Jebsen Center for Translational Research in Parkinson’s Disease, University of Bergen, 5020 Bergen, Norway

**Keywords:** Transporters in the nervous system, Drug screening, Protein structure predictions

## Abstract

Vesicular monoamine transporter 2 (VMAT2) is responsible for packing monoamine neurotransmitters into synaptic vesicles for storage and subsequent neurotransmission. VMAT2 inhibitors are approved for symptomatic treatment of tardive dyskinesia and Huntington’s chorea, but despite being much-studied inhibitors their exact binding site and mechanism behind binding and inhibition of monoamine transport are not known. Here we report the identification of several approved drugs, notably β2-adrenergic agonists salmeterol, vilanterol and formoterol, β2-adrenergic antagonist carvedilol and the atypical antipsychotic ziprasidone as inhibitors of rat VMAT2. Further, plausible binding modes of the established VMAT2 inhibitors reserpine and tetrabenazine and hit compounds salmeterol and ziprasidone were identified using molecular dynamics simulations and functional assays using VMAT2 wild-type and mutants. Our findings show VMAT2 as a potential off-target of treatments with several approved drugs in use today and can also provide important first steps in both drug repurposing and therapy development targeting VMAT2 function.

## Abstract (web summary)

Several existing β2-adrenergic agonists and antagonists, as well as the atypical antipsychotic ziprasidone, are identified as high affinity VMAT2 inhibitors, which could have implications for drug repurposing and drug derivatization campaigns.

## Introduction

In mammals, packing of monoamine neurotransmitters into vesicles is performed by the vesicular monoamine transporter 1 (VMAT1/SLC18A1) and vesicular monoamine transporter 2 (VMAT2/SLC18A2) of the solute carrier (SLC) 18 transporter family. VMAT1 and VMAT2 share a sequence identity of approximately 65% (Supplementary Fig. 1), but their expression profile differs. VMAT1 is mainly expressed in neuroendocrine cells in the sympathetic- and peripheral nervous system (SNS and PNS) and to some extent also in the central nervous system (CNS)^1–3^, while VMAT2 is mainly expressed in neurons in the CNS, in the SNS and in insulin-producing β-cells in the pancreas^4–6^. Monoaminergic dysfunction is involved in development of several neurological and psychiatric disorders such as depression, Parkinson’s disease (PD), Alzheimer’s disease, Huntington’s disease, attention-deficit hyperactivity disorder, and autism spectrum disorders, and can also be the result of long-term drug abuse^7^. VMAT function is important in dopaminergic neurons for packing of cytosolic dopamine (DA) into synaptic vesicles and subsequent neurotransmission^8,9^. Several studies connect specific VMAT1 variants to psychiatric disorders^7,10–12^ while VMAT2 function has been connected to PD development, as a larger reduction in VMAT2 activity compared with the reduction of dopaminergic neuronal markers has been measured in samples from PD-patients^13^. Dopaminergic neurons in the *substantia nigra* have high oxygen metabolism, low levels of antioxidants and high iron content, causing these neurons to be prone to oxidative stress^14^. High cytosolic DA levels can generate toxic reactive oxygen species (ROS) and DA-quinones which have been associated with the development of both PD and other neurodegenerative disorders^15,16^. The packing of monoamine neurotransmitters into synaptic vesicles by VMAT2 thus serves a dual purpose, being necessary for regulated exocytosis of neurotransmitters into the synaptic cleft, but also as a storage mechanism removing the reactive and potentially cytotoxic monoamine neurotransmitters from the cytoplasm^9,17,18^. Functional VMAT2 is also important in the pancreas, where it contributes to modulate insulin release by regulating dopamine levels and protects pancreatic β-cells from dopamine associated ROS cytotoxicity^19^.

**Fig. 1:**
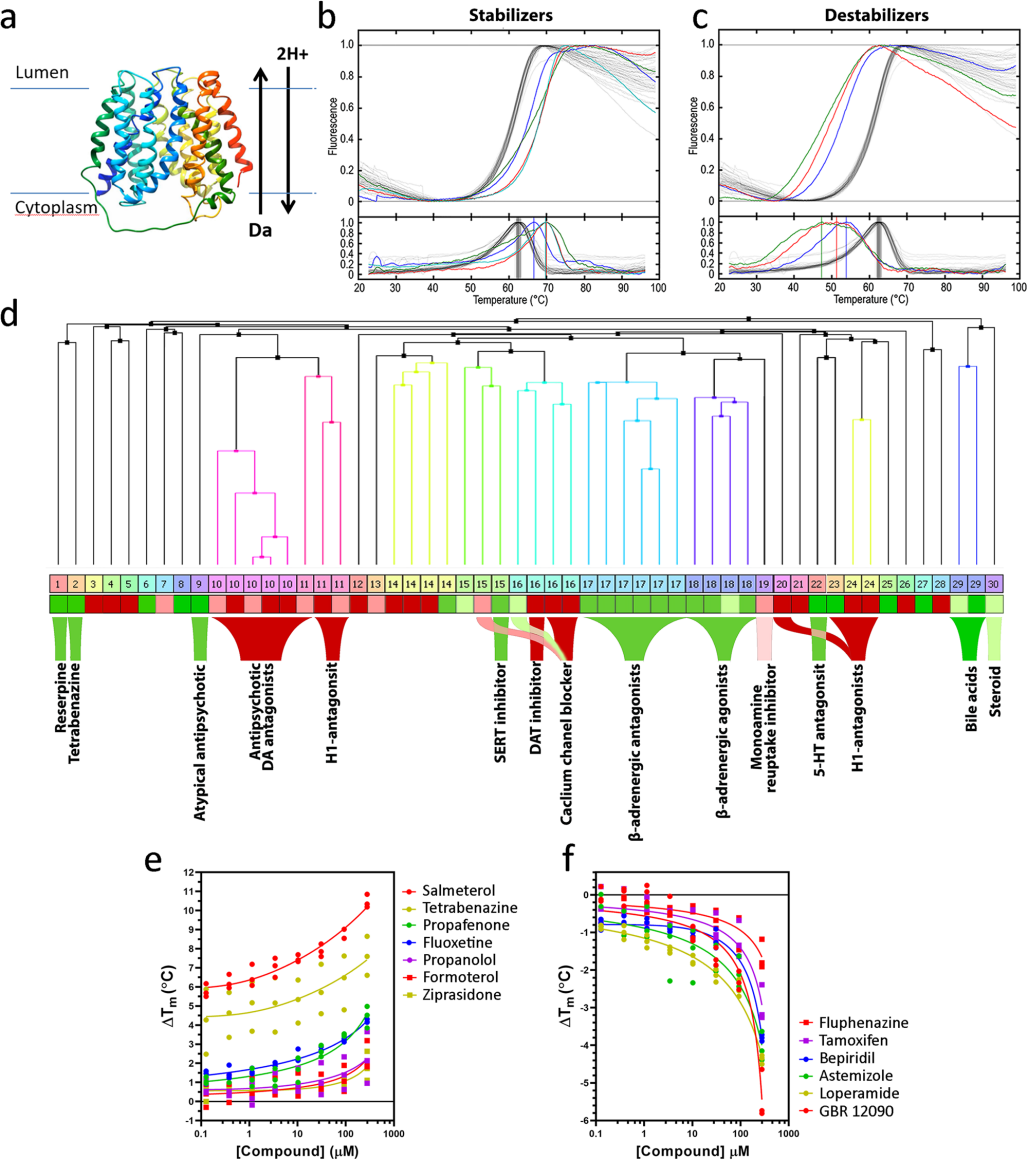
Screening for stabilizing or destabilizing compounds of VMAT2 by DSF. **a** Homology model of VMAT2 showing all 12 transmembrane α-helices. **b**, **c** Thermal melting curves from primary screening monitored by DSF at 167 µM in 1.67% DMSO (upper traces) and first derivative (bottom traces) with stabilizing compounds ziprasidone (ZPS) (blue), propafenone (red), tenatoprazole (green) and ketanserin (cyan), associated with increased T_m_ of VMAT2 (**b**) or with destabilizing compounds GBR 12909 (green), bepridil (blue) and lomerizine (red), that cause decreased T_m_ of VMAT2 (**c**). Gray curves are DMSO controls (1.67% DMSO). **d** Hierarchical clustering of compounds based on structural similarity (binary fingerprints). Clustering was performed using Schrödinger Canvas, and clustered compounds divided into 30 clusters for visualization. **e**, **f** ΔT_m-_values for VMAT2 incubated with the indicated stabilizing (**e**) and destabilizing (**f**) compounds at concentrations ranging from 120 nM to 270 μM.

VMAT2 has 12 transmembrane α-helices, organized in two six-helix bundles spanning the membrane (Fig. 1a)^20–22^. It is a secondary active antiporter, using the vesicular proton gradient generated by the V-type ATPase to transport monoamines against the concentration gradient into synaptic vesicles using an alternating-access mechanism^1^. The transition back to the cytoplasm-facing state is initiated by protonation of one or both carboxylic residues in the transmembrane region of helices TM1 (D33) and TM7 (E313), which are exposed in the central cavity of the transporter^23^. These residues are essential for transport of the anionic VMAT2 substrates and have been proposed to bind them directly^22^.

There are two main groups of approved VMAT inhibitors that block DA uptake into vesicles, leading to depletion of amine stores via DA degradation by cytosolic monoamine oxidase and reduction of monoamine neurotransmission^24^. Reserpine (RSP) is a high-affinity competitive inhibitor of both VMAT1 and VMAT2. It has been used as an antipsychotic, as an antihypertensive and for symptomatic treatment of chorea associated to Huntington’s disease^25,26^ but due to its adverse side effects it is seldom used today. A second group of VMAT2 inhibitors, tetrabenazine (TBZ) and the closely related deutetrabenazine (DEU) and valbenazine (VBZ), are non-competitive VMAT2 specific inhibitors that have a much lower affinity for VMAT1^20^. TBZ, DEU, and VBZ have been approved by the FDA for symptomatic treatment of tardive dyskinesia (TD) while TBZ and DEU have been used for symptomatic treatment of chorea associated with Huntington’s disease^24,27^. Identification of new inhibitors of VMAT2 thus has potential as treatment for TD and Huntington’s chorea. There are also VMAT2 specific inhibitors in development for treatment of amphetamine and methamphetamine abuse by reducing methamphetamine-evoked DA-release and in this way decreasing reward and abuse liability^28–30^. Moreover, non-inhibitory stabilizers and activators of VMAT2 have been suggested as treatment for patients with DA deficiencies, including PD^9,24,31^. There is thus a clear need for novel and more efficient inhibitory strategies, as well as interest in identifying compounds that can activate or increase VMAT2 function. Further, despite being well studied inhibitors, the exact binding sites and molecular mechanisms behind RSP and TBZ inhibition of VMAT1 and VMAT2 are not known.

To identify novel inhibitors or activators, we purified VMAT2 and screened a drug library containing 1280 off-patent drugs for binders with either stabilizing or destabilizing effect on the protein using differential scanning fluorimetry (DSF). Validated hit compounds were assayed for effect on VMAT2 activity in a cellular uptake assay using the fluorescent VMAT2 substrate FFN206^32^. Two inhibitory hits, the long-acting β-adrenergic receptor agonist (LABA) salmeterol (SMT) and the atypical antipsychotic ziprasidone (ZPS), were further investigated by molecular docking, MD-simulations as well as site-directed mutagenesis and additional binding assays together with the established VMAT2 inhibitors RSP and TBZ. Our work identifies several new VMAT2 inhibitors with nanomolar affinity, characterized plausible binding modes of some of these compounds and reveals details for the binding of known VMAT inhibitors, such as several key residues at the plausible binding modes.

## Results

### Screening for VMAT2 binders

To screen purified VMAT2 by DSF we expressed the rat VMAT2 protein, as VMAT2-His, in SF9 insect cells using the baculovirus expression system. The transporter was solubilized and purified in n-Dodecyl-β-D-maltoside (DDM)/cholesterol hemisuccinate (CHS) micelles by immobilized metal affinity chromatography (IMAC) and size exclusion chromatography (SEC). We screened the Prestwick Chemical Library® (PCL) containing 1280 off-patent drugs, most FDA- and EMA-approved and TBZ, which is not in the library, was included as a positive control. Screening was performed using DSF and the thiol-reactive dye 7-diethylamino-3-(4-maleimidophenyl)-4-methylcoumarin (CPM), an approach that monitors changes to the protein melting temperature (T_m_) upon compound binding^33^. Using CPM or other dyes, DSF-based assays have successfully been used in molecular-based screenings^34,35^. VMAT2 in 50 mM MES pH 6.5, 150 mM NaCl, 0.05% DDM and 0.005% CHS with 1.67 % DMSO (DMSO control) had a T_m_ = 62.6 ± 0.32 ^o^C (mean ± standard deviation (SD)) (Fig. 1b, c). The *initial hits* (binders) from the screening (59 compounds) were selected based on their increasing or decreasing effect on the T_m_-value (ΔT_m_) for VMAT2, and then subjected to a second DSF experiment at 200 µM compound (in 2% DMSO), in triplicate samples with a hit cut-off of │ΔT_m_│ = 5 x SD of the T_m_ of the DMSO control. Despite the rather stringent threshold value, we had 26 stabilizing (+ΔT_m_) and 28 destabilizing (-ΔT_m_) compounds *as primary hits* (Fig. 1b, c and Supplementary Table 1). The PCL included the known VMAT2 inhibitor RSP and together with TBZ these were 2 out of the 3 most effective VMAT2 stabilizers, with ΔT_m_ of 13.72 ± 0.05 and 8.17 ± 0.17 °C, respectively (Table 1). Moreover, other drugs previously shown to inhibit VMAT2^36,37^ such as fluoxetine, a selective serotonin transporter reuptake inhibitor (SSRI), and ketanserin, a DA/5-HT/α-adrenergic receptor antagonist, were also identified as VMAT2 stabilizers (Table 1 and Supplementary Table 1), confirming the validity of our screening approach to identify VMAT2 binders.

**Table 1:** Validated primary hits of VMAT2 binders, with ΔT_m_-values from DSF screening, as well as IC_50_ values for VMAT2 inhibition (FFN206 uptake) and competition of [^3^H]-DTBZ binding in cells.

Name	Class	ΔT_m_ (^o^C)	IC_50_ (μM)	[^3^H]-DTBZ binding at 50 μM compound (%)
Hit compounds, known VMAT inhibitors				
Reserpine (RSP)^a^	CNS	13.72 ± 0.05	ND	7.02 ± 11.21
Tetrabenazine (TBZ)^a^	CNS	8.17 ± 0.14	0.037 ± 0.028	0.49 ± 2.7
Fluoxetine^b^	CNS	1.55 ± 0.14	1.985 ± 0.254	11.62 ± 14.61
Chloroquine^c^	Met	−0.37 ± 0.23	>10	85.11 ± 11.3^e^
Hit compounds, new binders				
Biperiden	CNS	1.32 ± 0.22	ND	79.88 ± 19.12
Ziprasidone (ZPS)	CNS	2.69 ± 0.12	0.039 ± 0.017	36.54 ± 7.71
Fluphenazine	CNS	−5.24 ± 0.24	ND	67.98 ± 6.55
Lomerizine	CNS	−7.51 ± 0.04	3.898 ± 1.688	113.14 ± 23.24
Astemizole	Aller	−7.04 ± 0.40	1.171 ± 0.573	20.65 ± 12.59
Salmeterol (SMT)	Resp	9.71 ± 0.22	0.053 ± 0.028	0.37 ± 2.83
Formoterol	Resp	1.87 ± 0.19	0.565 ± 0.314	17.54 ± 4.4
Propafenone	CV	4.53 ± 0.18	3.384 ± 1.865	60.66 ± 20.82
Fluvastatin	CV	4.98 ± 0.17	ND	80.16 ± 34.76
(R)-Propranolol	CV	2.63 ± 0.13	2.211 ± 0.156	86.76 ± 18.87
Tenatoprazole	Met	7.36 ± 0.41	>10	87.45 ± 8.53
Tamoxifen	End	−5.92 ± 0.47	ND	70.33 ± 17.41
Loperamide	GE	−7.10 ± 0.28	1.930 ± 1.301	56.01 ± 18.72
Expanded selection				
Clenbuterol	Resp/ NM	2.19 ± 0.13	>10	ND
Indacaterol	Resp	−1.26 ± 0.15	0.372 ± 0.195	31.65 ± 4.76^e^
Vilanterol	Resp	ND	0.047 ± 0.014	ND
Olodaterol	Resp	1.09 ± 0.08	4.753 ± 1.363	95.56 ± 18.65^e^
Acebutolol	CV	2.14 ± 0.05	>10	ND
Bisoprolol	CV	1.49 ± 0.05	3.173 ± 0.895^d^	ND
Betaxolol	CV	1.98 ± 0.15	6.854 ± 3.620^d^	ND
Oxprenolol	CV	2.51 ± 0.08	6.548 ± 3.494	ND
Carvedilol	CV	−2.54 ± 0.19	0.185 ± 0.157	3.22 ± 3.37^e^
Pronethalol	CV	3.74 ± 1.33	1.011 ± 0.300	ND

*CNS* Central Nervous System, *CV* Cardiovascular, *GE* Gastroenterology, *Resp* Respiratory, *All* Allergology, *End* Endocrinology, *NM* Neuromuscular, *ND* not determined.

^a^Hit RSP was present in the Prestwich Chemical Library® (PCL), but not TBZ, which was included as positive control.

^b^Hit fluoxetine was present in the PCL and has previously been described as a VMAT2 inhibitor^32^.

^c^Chloroquine is present in the PCL and did not alter VMAT2 T_m_ but was included in activity assays as an additional control of VMAT2 activity. Chloroquine inhibits VMAT2 indirectly through disruption of the vesicular proton gradient^32, 85^.

**Fig. 2:**
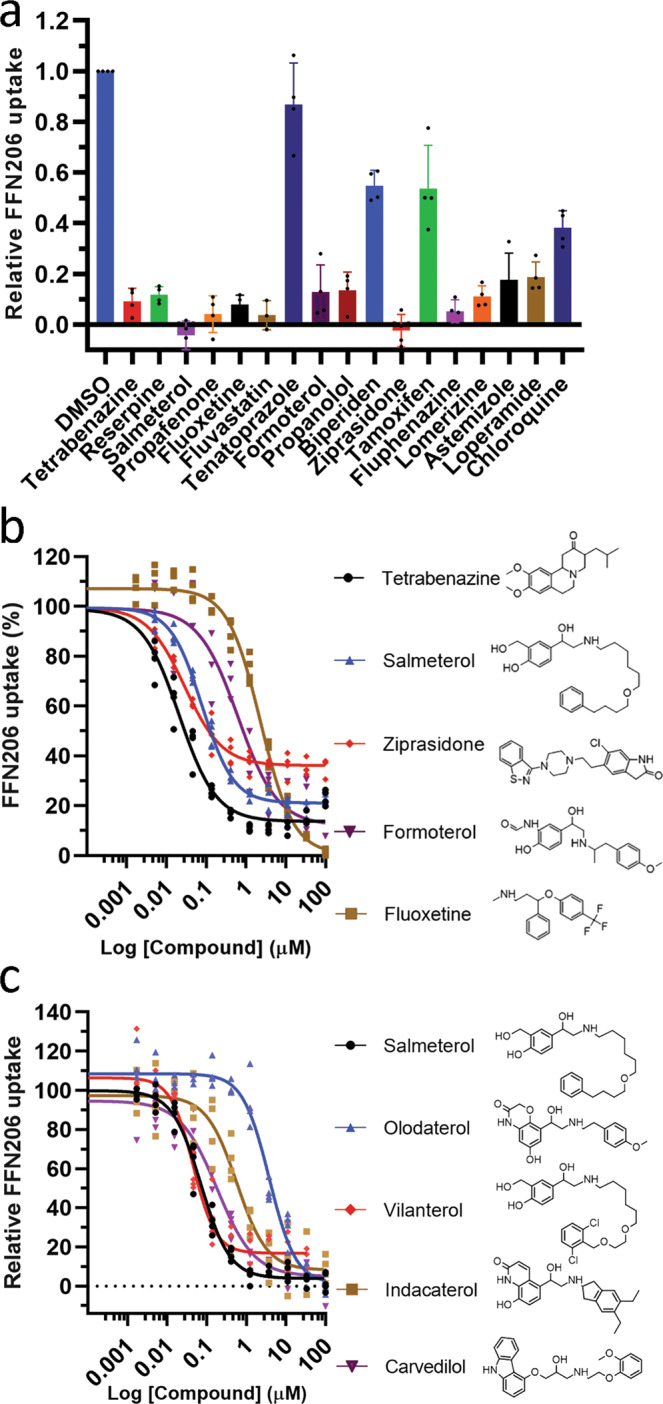
Inhibition of VMAT2 activity by hit compounds. **a** Inhibition of VMAT2-specific FFN206 uptake in transfected Hek293 cells. Bars represent mean ± SD, for 4 replicate samples for DMSO controls and each compound, at a final concentration of 25 μM. **b** Determination of the IC_50_-values for the inhibition of VMAT2 activity by tetrabenazine (TBZ, black), salmeterol (SMT, blue), ziprasidone (ZPS, red), formoterol (purple) and fluoxetine (yellow). **c** Determination of IC_50_-values for the inhibition of VMAT2 activity by salmeterol (SMT, black), and the β2-AR agonists and antagonists from the expanded hit selection olodaterol (blue), vilanterol (red), indacaterol (yellow) and carvedilol (purple). For **b**, **c**, data points from 3 or 4 replicate samples for each concentration of compound is shown. Calculated IC_50_ values for tested compounds are shown in Table 1.

IC_50_ values for inhibition of VMAT2 (FFN206 uptake) are provided as the mean ± SD of *n* ≥ 3 independent experiments with technical triplicates or quadruplicates (Fig. 2), except for ^d^bisoprolol and betaxolol where values from two independent experiments were aggregated and the calculated IC_50_ is given ±SEM from fitting of the curve.

The [^3^H]-DTBZ binding values are provided as the mean ± SD of *n* = 3 independent experiments with four technical repetitions. ^e^Compounds were tested for [^3^H]-DTBZ binding twice with four technical repetitions.

All primary hits were subjected to hierarchical clustering using FP2 fingerprints in Schrödinger Canvas, grouping compounds based on their structural similarities (Fig. 1d, Supplementary Table 1). Several serotonin-, DA-, and histamine-receptor antagonists (groups 9, 10, 11, 20, 23, and 24) and monoamine reuptake transporter inhibitors and calcium channel blockers (groups 15, 16, and 19) were identified as either stabilizers or destabilizers, while several β2-AR antagonists and agonists (groups 17 and 18) were found to stabilize VMAT2 (Fig. 1d, Supplementary Table 1). There were also some stabilizing steroidal drugs (group 29–30), which are structurally similar to cholesterol and also to CHS, which stabilizes detergent-solubilized integral membrane proteins, such as in our VMAT2 preparation. Further, we investigated the concentration dependent stabilization or destabilization of VMAT2 (concentration range 120 nM–270 µM), for all primary hit compounds by DSF. Most compounds had an effect at higher concentrations (90–270 μM), inducing no or very small ΔT_m_ below 90 μM (Supplementary Table 2), but several showed a statistically significant effect also at lower compound concentrations (Supplementary Table 2). Dose-response curves (ΔT_m_ vs compound concentration) for a selection of stabilizers and destabilizers are shown in Fig. 1e, f, respectively. After the concentration-dependent validation of the primary hits, 30 medium-high affinity binders (validated hits) remained (Supplementary Table 2), appearing as interesting candidates for functional studies.

### Several hit compounds are potent inhibitors of VMAT2

The functional effect of hit compounds was evaluated by cellular assays measuring substrate uptake and competition of [^3^H]-DTBZ inhibitor binding. Compounds that entered these studies were selected from the validated hits in Supplementary Table 2. Compounds that have had their FDA-approval withdrawn due to adverse side effects were filtered out. Moreover, for structurally very similar compounds, as determined by distances in the hierarchical clustering (Fig. 1d, Supplementary Table 1), only one was selected, resulting in a final selection of 13 binders, not previously connected to VMAT2, for functional assays (Supplementary Fig. 2, Table 1). As positive controls, we included the known VMAT2 inhibitors RSP and TBZ, and the SSRI fluoxetine. Moreover, chloroquine, which has previously been shown to inhibit VMAT2, however not through a direct interaction with VMAT2 but rather by disrupting the vesicular proton gradient that is generated by V-type ATPase^38^, was also tested (Table 1). Indeed, chloroquine did not affect the T_m_ of VMAT2 in our DSF-screen (Table 1), but it was still included in our VMAT2 activity assays as a control.

The effect of compounds on VMAT2 activity was tested first by uptake assays in transiently transfected Hek293 cells using the fluorescent VMAT2 substrate FFN206^32^. At relatively high compound concentrations (25 µM) several hits clearly inhibited VMAT2 activity (Fig. 2a), and their half-maximal inhibitory concentration (IC_50_) was determined (Fig. 2b, and Table 1). Tenatoprazole, biperiden and tamoxifen showed ≤50% inhibitory effect and were thus not subjected to IC_50_ determination. Two hit compounds, SMT and ZPS had IC_50_ values of 53 ± 28 and 39 ± 17 nM, respectively, a potency comparable to that of the specific VMAT2 inhibitor TBZ, i.e., IC_50_ = 37 ± 28 nM in our assay. The IC_50_ measured for TBZ, and the SSRI fluoxetine (1.98 ± 0.25 μM) is comparable to previously determined IC50 values^32,37,39,40^. Formoterol, a β_2_-AR antagonist, had an IC_50_ of 0.56 ± 0.31 μM and several other stabilizing and destabilizing compounds showed inhibition in the range 1–4 μM (Table 1). Chloroquine also inhibited VMAT2 mediated substrate uptake in our assays, but at much higher concentrations compared to the most potent hit compounds. As several β2-AR agonists and antagonists were among the most potent inhibitory hits, we expanded the selection with commercially available β2-AR agonists and antagonists. Several of the tested ultra-long acting β-AR agonist (ULABA) (olodaterol, indacaterol and vilanterol) and the β2-AR antagonist carvedilol, inhibited VMAT2 in the nM to low μM range (Fig. 2c). One of these, vilanterol, with similar structure to SMT (Fig. 2c), also presented a similar IC_50_ (47 ± 14 nM) as SMT and TBZ (Table 1).

**Fig. 3:**
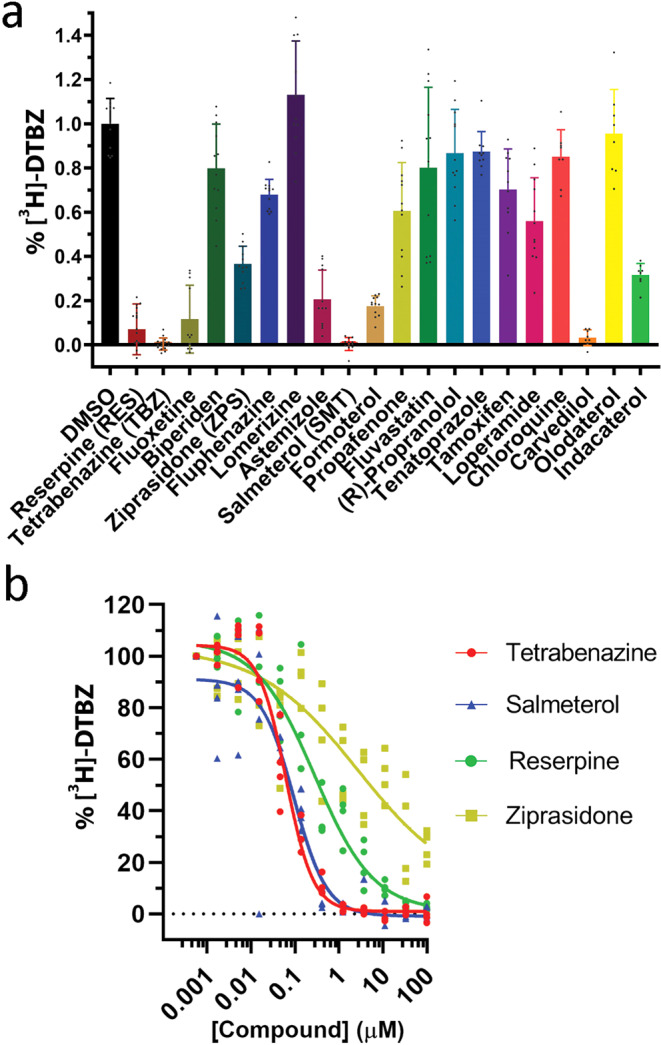
Competition of [^3^H]-DTBZ binding assay. **a** Cells were preincubated with 50 μM of compounds in 96 well plates and [^3^H]-DTBZ (5 nM) binding was measured. Bars represent mean ± SD, from 3 independent experiments with 3 or 4 repetitions for DMSO controls and each compound. Reserpine (RES), tetrabenazine (TBZ), fluoxetine, ziprasidone (ZPS), fluphenazine, astemizole, salmeterol (SMT), formoterol, propafenone, tamoxifen, loperamide, carvedilol and indacaterol had a statistically significantly effect on [^3^H]-DTBZ binding (compared with DMSO control; one-way ANOVA, *p* < 0.001). Biperiden, lomerizine, fluvastatin, propranolol, tenatoprazole, PCIPYR-0044, chloroquine and oldaterol had no statistically significant effect on [^3^H]-DTBZ binding. **b** EC_50_ determination for competition of [^3^H]-DTBZ binding by tetrabenazine (TBZ, red), salmeterol (SMT, blue), reserpine (RSP, green) and ziprasidone (ZPS, yellow).

Further, we examined the binding of compounds to VMAT2 using a competition of inhibitor binding assay with ^3^H labeled dihydrotetrabenazine ([^3^H]-DTBZ). We preincubated cells expressing VMAT2 with 50 μM compounds and measured [^3^H]-DTBZ binding (5 nM) to the sample (Fig. 3a). Not surprisingly, preincubation with 50 μM unlabeled TBZ, included as a positive control, completely hindered binding of 5 nM [^3^H]-DTBZ, while RSP, SMT and carvedilol all hindered [^3^H]-DTBZ binding by >90% (Fig. 3a, Table 1). Another LABA, formoterol, the ULABA indacaterol, fluoxetine, astemizole and ZPS all lowered [^3^H]-DTBZ binding by 60–85% (Fig. 3a, Table 1). Further, we determined the half-maximal effective concentration (EC_50_) for competition (inhibition of [^3^H]-DTBZ binding) for TBZ (60 ± 1.9 nM) and RSP (318 ± 35 nM), and for the hit compound SMT (85 ± 8.3 nM) (Fig. 3b, Table 1). For ZPS we clearly observed competition with [^3^H]-DTBZ binding, but a reliable EC_50_ could not be accurately determined (≤2 μM) (Fig. 3b).

Altogether these data show that several hit compounds inhibit VMAT2-mediated substrate uptake in transfected Hek293 cells. Of these, SMT, vilanterol and ZPS are potent inhibitors of VMAT2 with similar IC_50_ values as TBZ. Carvedilol and formoterol, both containing a similar ethanolamine group as SMT, also inhibit VMAT2 but with a 3.5-fold and 11-fold higher IC_50_ than SMT (Fig. 2b, c, Table 1). All these hit compounds also compete with [^3^H]-DTBZ for binding, suggesting that they directly interact with VMAT2.

### Molecular modeling and molecular dynamics simulations

To further examine how the VMAT2 inhibitors TBZ and RSP, as well as one of the β2-AR agonists, SMT, and the atypical antipsychotic ZPS bind to and inhibit VMAT2 function, we applied docking and MD-simulations. We used two homology models of the rat VMAT2 structure previously published and deposited to the Protein Model Database (PMDB); one cytoplasm-facing, VMAT2_CYT_ (PMDB id: PM0078823)^22^, and one lumen-facing model, VMAT2_LUM_ (PMDB id PM0080553)^23^. VMAT2 from rat and human have 91% sequence identity, with the transmembrane region and central cavity almost fully conserved (Supplementary Fig. 1). As it is difficult to predict which ligand state of each inhibitor binds to which VMAT2 state, we set up 20 different inhibitor:VMAT2 combinations through molecular docking of all predicted protonation states of the inhibitors to both VMAT2_LUM_ and VMAT2_CYT_ states (Supplementary Table 3). After stabilization of binding modes in MD simulations, we used implicit membrane MM/PBSA (IM-MMPBSA) to calculate the binding enthalpy for each complex. As described in the methods section, binding free energy is not computed, as the calculations do not include the entropic term.

**Fig. 4:**
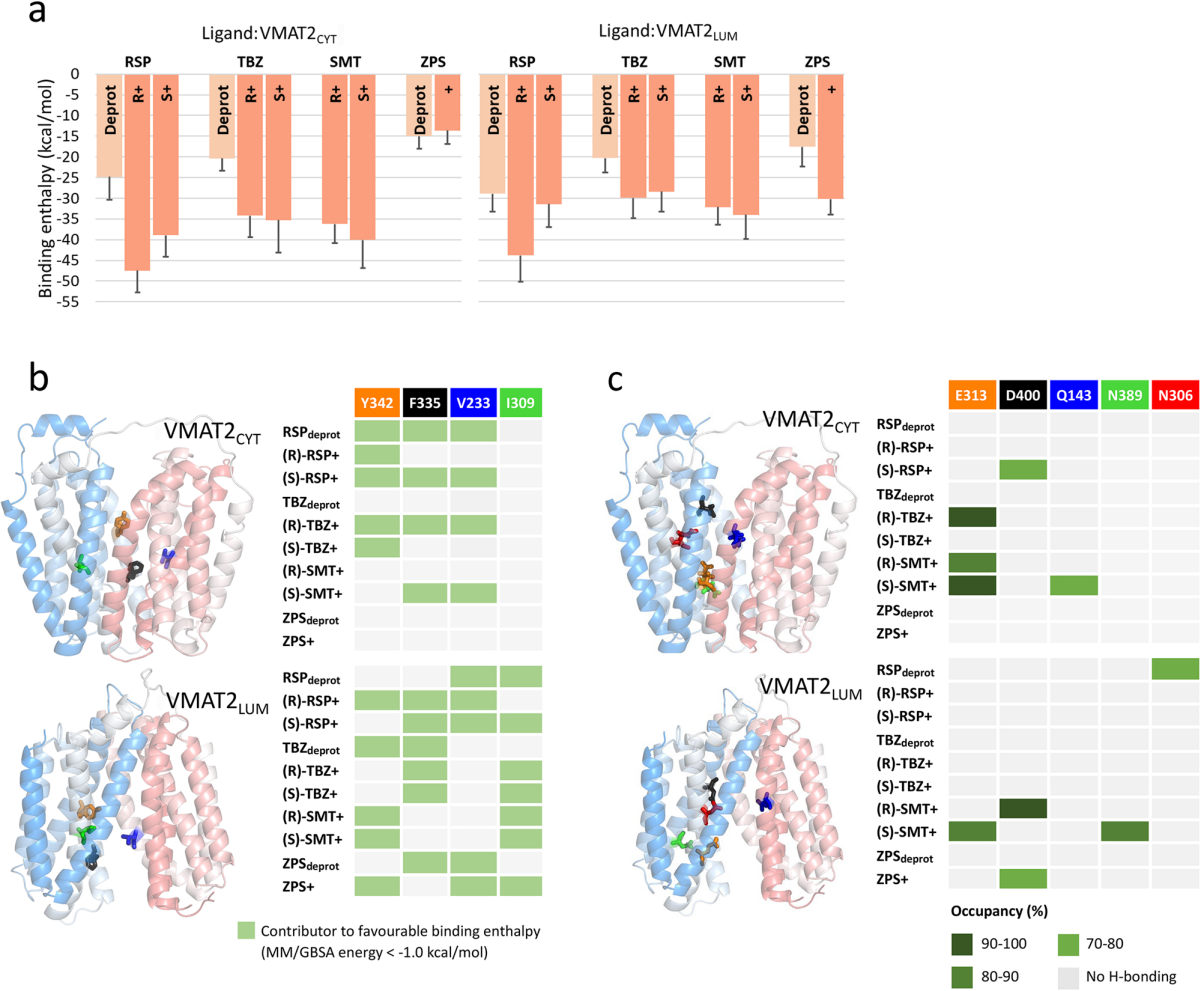
Binding enthalpies and “hot spot” VMAT2 residues from MD simulations. **a** Implicit membrane MM/PBSA binding enthalpies calculated from simulations of all ligand states, for reserpine (RSP), tetrabenazine (TBZ), salmeterol (SMT) and ziprasidone (ZPS) bound to the cytoplasm-facing VMAT2 model (left) and the lumen-facing VMAT2 model (right), and displayed as light colored bars for neutral, deprotonated ligand states and as dark orange bars for the protonated forms. Bars represent the average calculated binding enthalpy ±SD, calculated from 1000 frames from the last 200 ns of each simulation. **b** “Hot spot” residues. The four VMAT2 residues most frequently found among the main contributors to favorable enthalpy across all the simulation systems, along with their locations in the cytoplasm-facing (top) and lumen-facing (bottom) rat VMAT2 homology models, with VMAT2 colored by sequence from the N-terminal in red to the C-terminal in blue. Main contributors for each system were defined as residues with a more favorable decomposed MM/GBSA energy than −1.0 kcal/mol. Consult Table 2 for a more comprehensive overview. **c** Hydrogen bonding. Ligand:VMAT2 interaction partners displaying one or more hydrogen bonds present for more than half of the simulation time, i.e., hydrogen bonds with occupancies higher than 50%, and the locations of the relevant VMAT2 residues in the cytoplasm-facing (top right) and lumen-facing (bottom right) homology models. Consult the 2D ligand interaction diagrams in Supplementary Fig. 3 for more details.

To identify the VMAT2 residues that contributed most favorably to the ligand binding enthalpy in each simulation, we performed per-residue MM/GBSA energy decomposition. The residues displaying a decomposed energy contribution more favorable than −1.0 kcal/mol were subsequently extracted in ascending order for each simulation system, thereby indicating the most important residues for binding (Table 2). The resulting enthalpies are generally more favorable for positively charged forms compared to deprotonated forms, indicating that a balance of hydrophobicity and charge is important for ligand binding to VMAT2 (Fig. 4a). The calculated enthalpies also indicate that ZPS preferentially binds in its protonated form to VMAT2_LUM_. (R)-RSP^+^ displays the most favorable binding enthalpy in both VMAT states, and RSP enthalpies are generally more favorable relative to TBZ. Qualitatively, this correlates well with RSP having higher experimental VMAT2 binding affinity than TBZ^36^.

**Table 2:** MM/GBSA per residue decomposition.

VMAT2 state	Ligand state	Main contributors to favorable binding enthalpy^a^
VMAT2_CYT_	RSP_deprot_	I396, L229, V233, F335, Y342
	(R)-RSP^+^	Y342, L37, M205, I396, A338, S339
	(S)-RSP^+^	V233, Y342, L229, F430, I396, F335, L226, T38, A230
	TBZ_deprot_	M311, F136, F450, G133, A315, I318, P438, V132
	(R)-TBZ^+^	E313, Y342, F335, A338, V233, S339, A230
	(S)-TBZ^+^	Y342
	(R)-SMT^+^	E313, N389, K139, Y434, L316, V392, F430
	(S)-SMT^+^	E313, Y434, P238, L331, P317, V233, Q143, P237, F430, F335, L316, K139
	ZPS_deprot_	F136, P314, A315, I318, F450, M311, V132, N129, P438, Y434
	ZPS^+^	F136, M311, F450, P314, A315, I318, A445
VMAT2_LUM_	RSP_deprot_	K328, Y434, N306, V233, T38, L37, I309, W329, F430
	(R)-RSP^+^	L226, F430, A230, L229, F335, G397, Y342, F394, V233, M205, N34, M404
	(S)-RSP^+^	F335, V233, I309, L331, L229
	TBZ_deprot_	P317, F390, Y342, F335, M320, A338, L316, M321
	(R)-TBZ^+^	I309, F335, L316, P317, A310
	(S)-TBZ^+^	F335, L234, I309
	(R)-SMT^+^	D400, P314, Y342, A310, L343, T346, N306, F136, I309, G397, P438, A230
	(S)-SMT^+^	E313, Y342, N389, G393, I309, I396, I368, V392, F390
	ZPS_deprot_	F335, L331, V233, L336, P317, G332
	ZPS^+^	V233, Y342, A230, I309, I396, L226, Y434, D400

^a^VMAT2 residues with decomposed MM/GBSA energy contributions more favorable than cut-off of −1.0 kcal/mol sorted in ascending order, i.e., the top contributor is listed first.

**Fig. 5:**
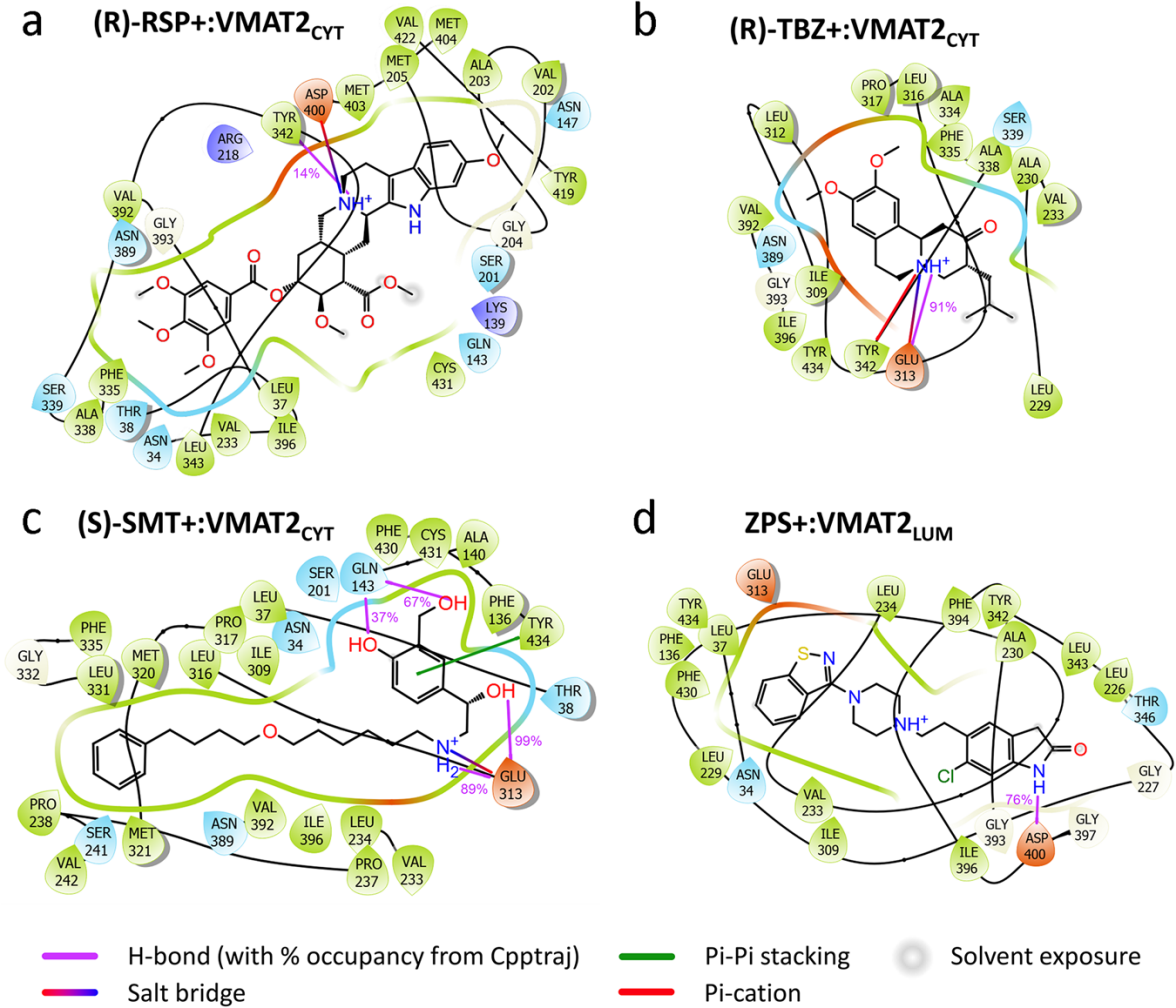
Representative ligand:VMAT2 binding modes from MD simulations. **a** (R)-RSP+:VMAT2_CYT_, **b** (R)-TBZ+:VMAT2_CYT_, **c** (S)-SMT+:VMAT2_CYT_ and **d** ZPS+:VMAT2_LUM_ provide the representative binding modes from the stable, last 200 ns portions of the ligand:VMAT2 simulations, as 2D interaction diagrams displaying the binding pockets defined by the VMAT2 residues within 4 Å of the ligand. The representative structure in each case was extracted as the centroid of the largest cluster following a clustering in CPPTRAJ based on the ligand position in the already RMSD-fitted protein. The 2D ligand interaction diagrams were generated using Schrödinger Maestro, and hydrogen bond occupancies calculated from the simulations using CPPTRAJ were added to the diagrams.

Y342, F335, and V233 stand out as the most frequently recurring residues among the main contributors to favorable binding enthalpy across all 20 simulation systems and are involved in ligand interaction in both VMAT2 states (Fig. 4b). Previous mutagenesis studies indicate that these three “hot spot residues” are important for VMAT function (Supplementary Table 4)^22,23^. I309 is another VMAT2 residue frequently found among the main contributors to favorable binding enthalpy, albeit only in ligand:VMAT2_LUM_ systems (Fig. 4b), indicating that this residue is possibly involved in binding the inhibitors at the lumen facing state. The ligand:VMAT2 interaction partners that engage in hydrogen bonds more than half of the time are shown in Fig. 4c, and the simulations indicate that E313 and D400 overall are the most important hydrogen bond participants. E313 is fully conserved in higher organisms, highly conserved in bacteria, and is crucial for VMAT2 activity^22^. D400 has been suggested to form a specific functional interaction with Y342 that is required for the transport of neurotransmitters in VMAT2^22^. Representative binding modes from all simulations are provided (Supplementary Fig. 3), and one example for each inhibitor is shown in Fig. 5.

### Binding modes of VMAT2 inhibitors RSP and TBZ

It has previously been shown that RSP binding is accelerated by the presence of a proton gradient, suggesting that RSP mainly binds to the cytoplasm-facing state^1^, although our MD simulations showed relatively high favorable binding enthalpies both in VMAT2_CYT_ and VMAT2_LUM_ binding modes. Y342 and D400 are contributing to sporadic hydrogen bonding, mainly in RSP:VMAT2_CYT_ simulations (Supplementary Fig. 3a, e), but overall, the main contributors to favorable enthalpy across all RSP:VMAT2 systems are dominated by hydrophobic residues (Table 2). This might reflect a largely hydrophobic binding site, as has been deduced from studies on bovine VMAT2 (bVMAT2)^41^. In the RSP:VMAT2_CYT_ systems, the trimethoxyphenyl portion inserts into approximately the same site (close to F335 and V233), with the rest of RSP in varying orientations (Fig. 5a, Supplementary Fig. 3a). Interestingly, the bVMAT2 structure-activity studies also indicated that trimethoxyphenyl provides a nonspecific contribution to the VMAT2 binding affinity^41^, and 2–4 (V233, F335, Y342, and I396) of the main enthalpic contributors pack around trimethoxyphenyl in each of the three stabilized RSP:VMAT2_CYT_ binding modes (Table 2 and Supplementary Fig. 3a).

TBZ binds to approximately the same region in VMAT2 in all six simulation systems, with at least part of the molecule in the interface between TM7, TM8, and TM10 helices, but in varying orientations and depths (Supplementary Fig. 3b, f). (R)-TBZ^+^:VMAT2_CYT_ is particularly interesting considering the high density of binding site residues (Fig. 5b and Table 2) with proposed roles in VMAT2 function and TBZ binding^22,23^. The protonated amine group of TBZ forms persistent and stable hydrogen bond and salt bridge interactions with the carboxyl group of E313, which will prevent E313 from carrying out its critical role in the VMAT2 transport cycle^22^. E313 is vital for both VMAT2 substrate transport and TBZ binding^22,23^. The rest of the inhibitor is subjected to tight hydrophobic packing involving residues V233, Y342, and F335, all of which have been shown to affect TBZ binding (Supplementary Table 4)^22^. In the simulations there is substantial overlap in binding site residues between the three TBZ:VMAT2_LUM_ systems, and with (R)-TBZ^+^:VMAT2_CYT_ and (S)-TBZ^+^:VMAT2_CYT_ (Supplementary Fig. 3b, f), while deprotonated TBZ stabilizes deeper and closer to the lumen side in the cytoplasm-facing model (Supplementary Fig. 3b). Of the previously discussed functional residues, F335 recurs as a main enthalpic contributor in the TBZ:VMAT2_LUM_ systems, as does Y342 in the case of TBZ_deprot_ (Fig. 4b and Table 2). Although it is not forming H-bonds in VMAT2_LUM_ simulations, E313 is also found in the VMAT2_LUM_ binding pockets, and can possibly form a salt bridge with the protonated amine in (S)-TBZ^+^(Supplementary Fig. 3f). In addition, I309 appears to be important for binding of the protonated forms in VMAT2_LUM_ judging by the per-residue decomposition.

### Ethanolamine is identified as a key player in SMT binding

Interestingly, overall binding enthalpies for all SMT-VMAT2 simulation systems were similar to all TBZ-VMAT2 systems, suggesting that SMT can bind as strongly to VMAT2 as TBZ (Fig. 4a). This finding is also in accordance with the similar ΔT_m_ and IC_50_ values for both compounds, and with results from binding competition with [^3^H]-DTBZ (Table 1). SMT displays the most prevalent hydrogen bonding among the simulated ligand:VMAT2 complexes (Figs. 4c and 5c). A signature pattern is replicated in all four systems, whereby the hydrogen bond potential of the protonated ethanolamine group in SMT is maximized through extensive and stable interaction with the carboxyl group of either E313 (in three of the systems; (R)-SMT^+^:VMAT_CYT_, (S)-SMT^+^:VMAT_CYT_ and (S)-SMT^+^:VMAT_LUM_) or D400 ((R)-SMT^+^:VMAT_LUM_) (Supplementary Fig. 3c, g). Its significance in the simulations is underlined by the prediction of E313/D400 as the top contributors to the favorable binding enthalpy in all four SMT systems (Table 2). It is worth noting that the ethanolamine group in SMT is a common denominator among all β2-AR agonists and antagonists in Table 1, some of which display submicromolar or near submicromolar IC_50_ values (vilanterol, indacaterol, formoterol, carvedilol, pronethalol), where the ethanolamine group might contribute to anchoring the inhibitor to VMAT2. The interaction pattern of the whole (S)-SMT^+^ phenylethanolamine moiety is similar in both VMAT states, with the E313 hydrogen bonding and the benzene ring engaging in pi-pi-like aromatic interaction (with Y434 in VMAT2_CYT_, F335 in VMAT2_LUM_), while its hydroxyl and hydroxymethyl substituents are involved in hydrogen bonds (with Q143 in VMAT2_CYT_, N389 in VMAT2_LUM_) (Fig. 5c and Supplementary Fig. 3c, g). This combined occurrence is less pronounced in the (R)-SMT^+^ systems, suggesting that the S configuration might be more suitable for anchoring the whole phenylethanolamine portion to VMAT2.

**Fig. 6:**
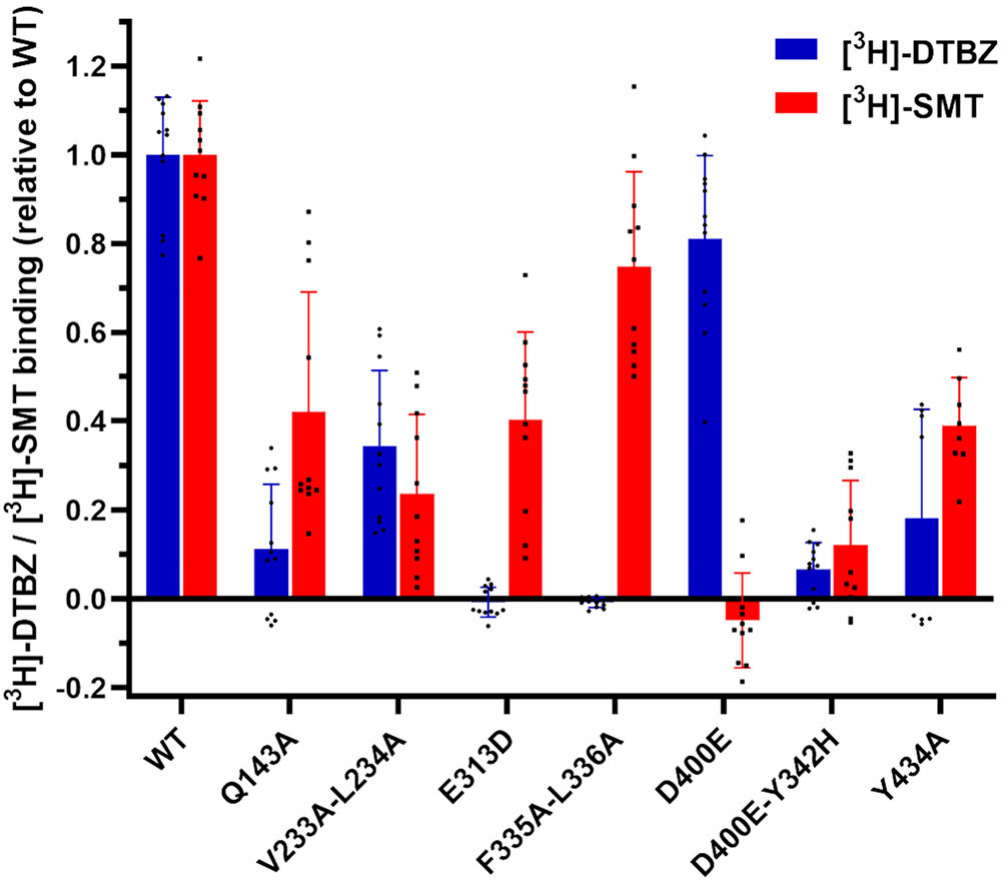
[^3^H]-DTBZ and [^3^H]-SMT binding to VMAT2 mutants. Rat VMAT2-Strep-tagII wild-type (WT) and mutants were expressed in Hek293 cells, solubilized from membranes in detergents and bound to Strep-Tactin Sepharose beads. VMAT2-Strep-tagII bound beads were transferred to 96 well filter plates and assayed for [^3^H]-DTBZ and [^3^H]-SMT binding. Bars show results from three independent experiments with either three or four replicates, Y434A was repeated twice with four replicates. [^3^H]-DTBZ and [^3^H]-SMT binding was correlated to the amount of VMAT2-Strep-tagII present in each sample, determined by SDS-PAGE and western blotting. Statistically significant differences from wild-type, as determined by one way ANOVA: Q143A, V233A-L234A, E313D, D400E-Y342H and Y434 mutants displayed differences in both [^3^H]-DTBZ and [^3^H]-SMT binding, *p* < 0.01. F335A-L336A abolished [^3^H]-DTBZ binding (*p* < 0.0001) and had a small but non-significant decrease in [^3^H]-SMT binding, while D400E abolished [^3^H]-SMT binding (*p* < 0.0001) and had a small but non-significant decrease in [^3^H]-DTBZ binding.

To further validate SMT binding to VMAT2 and to probe the involvement of residues identified as key contributors to binding enthalpy in TBZ and SMT binding modes, we created several VMAT2 mutants (listed in the methods section) and determined [^3^H]-DTBZ and [^3^H]-SMT binding to VMAT2, wild-type and a selection of mutants (Fig. 6). We prepared the mutants E313D, D400E, Q143A and Y434A, all in residues identified as important for hydrogen binding either directly to inhibitors or to E313 in different systems, notably for SMT (Fig. 4c and Supplementary Fig. 3c, g), and the D400E-Y342H mutant, involving residues that have been suggested to form an interaction that is necessary for transport^22^. Further, V233A-L234A and F335A-L336A also involve hot spot residues that work together as hinge points in VMAT2 transport^22^, and were identified as key contributors to binding of TBZ and SMT, but also RSP and ZPS (Table 2, Fig. 4b). [^3^H]-DTBZ and [^3^H]-SMT binding assays reveal that mutation of several residues identified as key contributors in binding modes, such as Q143A, V233A-L234A and Y434A, have a deleterious effect on the binding of both inhibitors, but especially for [^3^H]-DTBZ (Fig. 6). E313 was essential for [^3^H]-DTBZ and had reduced [^3^H]-SMT binding, and further F335A-L336A had no [^3^H]-DTBZ binding but retained [^3^H]-SMT binding. D400E on the other hand had a minimal effect on [^3^H]-DTBZ binding but appears essential for [^3^H]-SMT binding at the tested concentration (Fig. 6). Altogether these data validate SMT as a direct binder of VMAT2 and support key findings from docking and MD simulations.

### ZPS preferentially binds to the lumen-facing VMAT2 state

Based on the calculated binding enthalpies in Fig. 4a, ZPS preferentially binds in its protonated form to the lumen-facing VMAT2 state. Apart from extensive hydrogen bond interaction between the oxindole NH group and D400 in the ZPS^+^:VMAT2_LUM_ binding mode, the binding can be characterized by tight packing of hydrophobic residues around the ligand (Fig. 5d and Supplementary Fig. 3h). Y342 packs closely to the opposite edge of the oxindole ring from D400. V233, the top contributor to the negative enthalpy value, remains near one side of the benzothiazole ring of ZPS, while Y434 stacks parallel to and nearly on top of the other side of the ring. In this binding mode, ZPS would interfere with the D400-Y342 functional bond and the hydrophobic hinge cluster (V233, L234, F335, L336^22^), and at the same time interact closely with and potentially immobilize Y434 (Supplementary Fig. 3h). In the ZPS:VMAT2_CYT_ binding modes however, both binding modes (ZPS_deprot_:VMAT2 and ZPS^+^:VMAT2) overlap substantially with each other and with TBZ_deprot_:VMAT2_CYT_.

To validate ZPS binding to VMAT2 and substantiate the binding modes from MD simulations we analysed a selection of VMAT2 mutants and determined the IC_50_ values for inhibition of FFN206 uptake by ZPS, in comparison with TBZ and SMT (Table 3). As the binding models for ZPS binding have pointed to several key binding contributors (F136, V233, F335; Figs. 4c, 5d, Table 2 and Supplementary Fig. 3d, h) that have previously been shown to be important for substrate transport^22,42^ we prepared additional single and double alanine mutants (F136A, F136A-F335A, V233A-L234A, F335A, and F335A-L336A). However, none of these mutants had sufficient VMAT2 activity to reliably determine IC_50_ values in our assay (Table 3). We also prepared P314A which was among the main contributors to binding enthalpy in both ZPS:VMAT2_CYT_ binding modes (Table 2, Supplementary Fig. 3d) and included Q143A in our analysis, resulting in approx. 3- and 10-fold increased IC_50_ for ZPS inhibition, respectively (Table 3). Finally, Y434A had only residual substrate transport, which was inhibited by SMT, but not TBZ and ZPS (Table 3). Although an unambiguous confirmation of the MD-derived binding modes for ZPS could not be extracted from this mutagenesis study, the results are in accordance with direct binding of this inhibitor to VMAT2.

**Table 3:** IC_50_ for the inhibition of VMAT2 (measured as FFN206 substrate uptake) by tetrabenazine (TBZ), salmeterol (SMT) and ziprasidone (ZPS) for VMAT2 wild-type and selected mutants.

VMAT2 mutant	IC_50_ (μM)		
	TBZ	SMT	ZPS
Wild-type	0.037 ± 0.028	0.053 ± 0.028	0.039 ± 0.017
Q143A	0.366 ± 0.049	0.148 ± 0.033	0.354 ± 0.083
P314A	0.339 ± 0.172	0.234 ± 0.065	0.132 ± 0.008
Y434A	ND	0.126 ± 0.087	ND

ND not determined due to lack of inhibition within the range of tested concentrations (0–10 μM). VMAT2 mutants F136A, F136A-F335A, V233A-L234A, F335A, and F335A-L336A were tested in this assay but did not have sufficient activity or inhibition to determine a reliable IC_50_ value.

## Discussion

Using VMAT2 detergent-solubilized and purified from membranes, we screened the PCL by CPM-based DSF and identified new inhibitors of VMAT2. The validated hits included several compounds known to inhibit VMAT2 (TBZ, RSP, ketanserin, and fluoxetine), as well as DA receptor (D1, D2, and D3)-, serotonin receptor (5-HT1 and 5-HT2)- and histamine receptor (H1)-antagonists. Further, we found several β2-AR agonists and antagonists as stabilizers of VMAT2 and showed that several of these - SMT, vilanterol, formoterol, and carvedilol, as well as the atypical antipsychotic ZPS - are potently inhibiting VMAT2 activity. These monoaminergic receptor targeting drugs have, to the best of our knowledge, not been previously described as inhibitors of VMAT2.

To our knowledge, this work also presents the first MD simulations of VMAT2 and its complexes with the known inhibitors RSP and TBZ, that providing further insights into their binding and inhibitory mechanism. The VMAT2 homology models were built on distant bacterial homologs and should be treated with caution, however both the VMAT2_CYT_ and VMAT2_LUM_ models used are expected to have the reasonable backbone accuracies of 1.5–3.5 Å and 1–2 Å, respectively^22,23^, and were considered viable starting points for docking and MD simulations. The validity of the simulated structures is supported by the effect of site-directed mutations both on inhibitor binding (Fig. 6) and on the effect of inhibitors on substrate uptake (Table 3), as well as by previous experimental observations (Supplementary Table 4)^22,23,42,43^. In most of their binding modes RSP, TBZ and SMT stabilized deep in the central cavity of VMAT2 with similar binding enthalpies, and to some extent also with overlap in binding site residues in both the VMAT2_CYT_ and VMAT2_LUM_ systems (Table 2). In the VMAT2_LUM_ systems also ZPS bound deeper in the central cavity, with some overlap in binding residues with TBZ:VMAT2_LUM_ systems, while in VMAT2_CYT_ systems ZPS_deprot_ and ZPS stabilized in configurations close to the lumen side of VMAT2 with less favorable binding enthalpies. F335, V233, and Y342 contributed the most to the favorable binding enthalpy for TBZ-VMAT2_CYT_ and RSP-VMAT2_CYT_ modes, and all these residues, together with I309, were also important contributors to TBZ-VMAT2_LUM_, RSP-VMAT2_LUM,_ and ZPS-VMAT2_LUM_ binding enthalpies, and to some extent to SMT-VMAT2_LUM_. Contrary to SMT, RSP and ZPS, which inhibit both VMAT1 and VMAT2 (Supplementary Table 1 and ref. ^44^), TBZ is a specific VMAT2 inhibitor with only very weak inhibition of VMAT1. Several of the main contributors to favorable enthalpy across the TBZ simulation systems correspond to residues that are identical in VMAT2 from both rat and human but differ from their VMAT1 counterparts in residues V233, I309, M311, A315, and F390 (Supplementary Fig. 1). V233 and I309 are among the main binding contributors to all ligands, while A315 (T315 in VMAT1) and F390 (A390 in VMAT1) are mainly stabilizing TBZ and ZPS binding. Human and rat VMAT2 share a very high sequence homology (91% sequence identity, Supplementary Fig. 1), especially in the transmembrane region, and all the 57 different residues that were identified as top contributors, aggregated from all our 20 simulation set ups, are conserved between rVMAT2 and hVMAT2 (Table 2 and Supplementary Fig. 1). Indeed, TBZ, SMT and ZPS inhibition of human VMAT2 resulted in approximately the same IC_50_ results as for rat VMAT2 (Supplementary Table 1). Altogether these data thus suggest that even though most of our assays have been performed using rat VMAT2, the findings are relevant also for the human VMAT2 orthologue.

ZPS inhibited VMAT2 with a similar IC_50_ as TBZ, although it did not reach the same level of maximum inhibition as TBZ and SMT in our assays. ZPS also acted as a stabilizer of purified VMAT2, and could compete with DTBZ in VMAT2 binding, but more studies are needed to investigate the effects of ZPS on VMAT2 stability and activity in vivo. ZPS acts as a DA receptor antagonist and a partial agonist of the 5-HT1 and 5-HT2 receptors and is used in treatment of schizophrenia and manic episodes in patients with bipolar disorder. A common side effect among both typical and atypical antipsychotics, including ZPS, is drug-induced TD, a comorbidity which is currently treated with VMAT2 inhibitors^45,46^. Patients treated with ZPS present, as with most atypical antipsychotics, a lower incidence rate of TD development compared with typical antipsychotics, but there is no clear difference in incidence rate among ZPS and other atypical antipsychotics^47^.

β2-AR antagonists carvedilol, pronethalol and propranolol also inhibited VMAT2 function. Propranolol and carvedilol cross the blood-brain barrier (BBB) and have previously been suggested as treatment options for patients with drug-induced TD^48–50^. Propranolol has also been shown to reduce L-DOPA induced dyskinesia (LID) in humans^51^ and parkinsonian mice^52^. Although these studies ascribe the favorable effects of propranolol to β2-AR antagonism, the effects closely resemble what is seen for VMAT2-specific inhibitors in the treatment of TD^48–51^. Our results thus point to neuronal VMAT2 as a possible off target for the identified β2-AR antagonists and also suggest these drugs as starting points for further drug derivatization and as potential future treatment options for TD that should be explored further.

From the several β2-AR agonists identified as stabilizers of VMAT2, the two ULABAs SMT and vilanterol were highly potent VMAT2 inhibitors, with similar IC_50_ as TBZ for inhibition of substrate uptake. SMT also shows a low EC_50_ value for competition in the [^3^H]-DTBZ binding assay. This is in line with MD simulations showing comparable binding enthalpies for SMT and TBZ in each of the two VMAT states. SMT and TBZ also have partially overlapping binding modes both in VMAT2_LUM_ and VMAT2_CYT_ (Supplementary Fig. 3b, c, f, g). Similar to the (R)-TBZ+:VMAT2_CYT_ binding mode, the main factor in the binding of SMT to VMAT2_CYT_ in MD simulations was the stable hydrogen bond and salt bridge interaction between the carboxyl group of E313, or D400 in the (R)-SMT^+^-VMAT2_LUM_ binding mode, and the protonated ethanolamine group in SMT (protonated amine in TBZ). This could indicate that the ability of SMT to compete with [^3^H]-DTBZ binding relies on a strong interaction with E313 or D400. This ethanolamine group in SMT is present in all the β2-AR agonists and antagonists that were identified as VMAT2 stabilizers and is most likely an important contributor to the binding of these compounds to VMAT2. Although inhaled β2-AR agonists such as SMT and formoterol act locally in the lung, there is also systemic absorption across the pulmonary vascular bed and from the gastrointestinal tract due to the high gut deposition of inhaled drugs^53,54^. SMT and formoterol can also partially cross the BBB^55,56^ and can thus potentially affect VMAT1 or VMAT2 function both in the CNS and outside the brain. Our findings that β2-AR agonists inhibit VMAT2 may identify off-target effects of these medications, and thus highlight that the role of DA and VMAT2 function in possible treatment-associated dysfunctions, in and outside the brain, should be studied further.

In conclusion, we describe the identification of several FDA approved drugs as VMAT2 inhibitors which are interesting from a drug repurposing perspective aimed at for instance treatment of TD and Huntington’s chorea. These findings can also contribute to our understanding of off-target effects of these medications, although more studies are needed to examine the in vivo relevance of our results. By docking and MD simulations we have identified plausible VMAT2 binding modes of the investigated inhibitors that correlate well with experimental evidence from our mutagenesis analysis and results available in the literature. The simulations point to plausible binding modes for RSP, TBZ, SMT, and ZPS, identify residues important for inhibitor binding and indicate that the ethanolamine group is a key player in the binding of SMT (and potentially other β2-AR agonists/antagonists) to VMAT2. Altogether this information can prove useful in the continuing investigation into VMAT2 inhibitor binding and early-stage drug development efforts.

## Methods

### Protein expression and purification

Recombinant rat VMAT2-His was expressed in Sf9 insect cells using the baculovirus expression system. The ORF of rat VMAT2 was subcloned into a pFastBac vector and transformed into DH10Bac strain for bacmid generation. The transformed cells were grown in SOC medium for 4 h, allowing Tn7 transposition, plated on agar plates with 50 μg/ml kanamycin, 7 μg/ml gentamycin, 10 μg/ml tetracycline, 25 μg/ml X-gal and 40 μ/ml IPTG and positive colonies were selected by blue/white screening. Bacmid DNA was prepared by alkaline lysis and ethanol/isopropanol precipitation as described in ref. ^57^. Sf9 cells was transfected with isolated bacmid DNA and grown in 24 well plates for 7 days at 27 °C. Released virus was harvested and used to generate a high-titer viral stock which was used for protein expression. Sf9 cells were grown to a density of 2.0 × 10^^6^ cells/ml infected with baculovirus and harvested at day 3 after growth arrest. Harvested cells were pelleted in a Beckman Coulter Avanti J-20 centrifuge at 6000 g, resuspended, and washed in 1× PBS (pH 7.4), and pelleted again in 50 mL falcon tubes using a Beckman Coulter Allegra X-12 centrifuge. Harvested cells were lysed using French press and the insoluble fraction containing cell membranes pelleted at 180,000 g for 1 h in a Beckman Coulter Optima MAX-TL ultracentrifuge. Collected membranes were resuspended in resuspension buffer (50 mM MES pH 6.0, 150 mM NaCl, with protease inhibitors) and homogenized using a dounce homogenizer with 30–50 strokes and pelleted again at 180,000 g for 1 h. Pelleted membranes were resuspended and washed in resuspension buffer with high ionic strength (500 mM NaCl) to remove peripherally bound membranes, pelleted again at 180,000 g and finally resuspended in resuspension buffer (50 mM MES pH 6.0, 150 mM NaCl, with protease inhibitors containing 10% glycerol at a wet weight concentration of 200–500 µg/ml). For protein purification membrane pellets were thawed and diluted to a protein concentration of approximately 5 mg/ml (as determined by a Direct Detect spectrophotometer (Millipore)) and solubilized in 1% n-Dodecyl-β-D-maltoside (DDM) and 0.2% cholesterol hemisuccinate (CHS) for 1.5 h on a rotary wheel at 4 °C. Unsolubilized material was removed by ultracentrifugation at 180,000 g for 1 h, and the supernatant containing solubilized membranes was further diluted 1:1 in resuspension buffer without detergent and batch incubated with TALON affinity resin (TaKaRa) for 6 h at 4 °C. The resin was collected and washed with >20 column volumes of wash buffer (50 mM MES pH 6.0, 150 mM NaCl, 0.05% DDM, 0.005% or 0.01% CHS, 10 mM imidazole) and eluted by step elution in elution buffer (50 mM MES pH 6.0, 150 mM NaCl, 0.05% DDM, 0.005% or 0.01% CHS, 300 mM imidazole). Fractions from elution were analyzed by SDS-PAGE and fractions containing VMAT2 were pooled and concentrated in Amicon Ultra 100 kDa spin columns or Proteus X-spinner 2.5 (100 kDa MWCO, PES) to a final volume of approximately 600 μl. The concentrated protein sample was further purified by size exclusion chromatography (SEC) on a Superdex 200 increase 10/300 column connected to an AKTA Pure protein purification system. Fractions containing monomeric VMAT2 were collected and analyzed by SDS-PAGE (purity > 85%). SDS-PAGE analysis was done using 4–20% Mini-PROTEAN Precast gels from BioRad, and gels were imaged using a Chemidoc XRS system from Biorad and the purity was analyzed in Image Lab (BioRad).

### Compound screening by differential scanning fluorimetry (DSF)

DSF screening was performed in 384 well plates using the thiol-sensing dye 7-diethylamino-3-(4-maleimidophenyl)-4-methylcoumarin (CPM) (Merck). Purified protein (50–100 ng/μl) was mixed with 20 ng/µl CPM and compounds (120 nM–500 μM final concentration) and preincubated at room temperature for 20 min before DSF analysis. Protein melting curves were determined using a LightCycler 480 ii RT-PCR system (Roche), with a temperature ramp increasing from 25 to 95 °C at a rate of 2 °C/min. Data analysis was performed using an in-house software that automatically determines the protein midpoint melting temperature (T_m_) based on the second derivative of the melting curves or using the HTSDSF explorer as described in ref. ^58^. For the initial compound screening a master mix of purified VMAT2 (50 ng/μl final concentration) and CPM (20 ng/μl final concentration) in a buffer with final concentration of 50 mM MES pH 6.5, 150 mM NaCl, 0.025% DDM and 0.0025% CHS was prepared. 6 μl of this mix was dispensed to each well of 384 well plates using a Multidrop™ Combi Reagent Dispenser (Thermo Scientific). The 384 well plates were then transferred to a mosquito® HV system (TTP Labtech) and 4 μl of a compound/buffer mixture was added to a final concentration of either 500 or 167 μM compound and 5% or 1.67% DMSO, respectively. Compounds that either stabilized or destabilized VMAT2 with a ΔT_m_ > 5× the standard deviation (SD) of the DMSO controls in each plate were classified as initial hits. ΔT_m_ was calculated by subtracting the T_m_ of VMAT2 with compound from the T_m_ of VMAT2 DMSO control (ΔT_m_ = T_m(compound)_ - T_m(DMSO control)_. All initial hits were then subjected to an initial validation in triplicates with a compound concentration of 200 μM and a ΔT_m_ > 5× the standard deviation (SD) cut off. The resulting primary hit compounds were further subjected to a concentration dependent validation assay in which VMAT2 was mixed with the hit compounds at 8 different compound concentrations in the range 120 nM–270 μM (2.7% DMSO), in triplicates. In validation experiments the sample mix had a buffer with final concentration of 50 mM MES pH 6.5, 150 mM NaCl, 0.05% DDM and 0.01% CHS. Processing of the data from the primary screening and the concentration-dependent validation was performed as reported^58^.

### Preparation of VMAT2 expression plasmids

For activity assays, [^3^H]-DTBZ and [^3^H]-SMT binding and competition assays (see section below), several VMAT2 mutants were prepared. The plasmids used in these studies where pcDNA3.1 containing ORFs coding for rat (r) *VMAT2*-His wild-type (WT), human (h) *VMAT2*-His WT and h*VMAT1*-His WT. In addition, a selection of r*VMAT2* mutants carrying mutations designed from binding modes identified by docking and MD-simulation was prepared in a pcDNA3.1 r*VMAT2*-Strep tag II plasmid. The r*VMAT2* mutants included F136A, F136A-F335A, Q143A, V233A-L234A, P314A, E313Q, E313D, F335A, F335A-L336A, D400E, D400E-Y342H, and Y434A, all of which were expressed in Hek293 cells to determine the half-maximal inhibitory concentration (IC_50_) of the identified inhibitors.

### FFN206 uptake assays

FFN206 uptake assays were performed in 96 well plates using Hek293 EBNA cells transiently expressing rat VMAT2. Hek293 cells were grown in 10 cm dishes until approximately 50–70% confluence and transfected with pcDNA3.1 r*VMAT2*-His WT, h*VMAT2*-His WT and h*VMAT1*-His WT or pcDNA3.1 *VMAT2*-Strep-tag II WT or mutants using Lipofectamine 3000 or Lipofectamine LTX according to the manufacturer protocol. Untransfected Hek293 cells or Hek293 cells transfected with empty vector were used as controls. Cells were then incubated for 24 or 48 h, trypsinized, washed and reseeded into poly-L-lysine and poly-D-lysine coated Corning BioCoat 96 well plates at approximately 80% confluency. Each 96 well plate contained both VMAT2 transfected cells and Hek293 control cells that were used as controls to measure unspecific substrate uptake or signal background. The following day, media in the 96 well plates were exchanged using a multichannel pipette and the cells were preincubated in Opti-MEM medium containing compounds or DMSO controls. After 30 min of preincubation, the fluorescent VMAT2 substrate FFN206 diluted in Opti-MEM was added to the cells at a final concentration of 5 μM and incubated for 15 or 30 min at 37 °C, 5% CO_2_. FFN206 uptake was stopped by washing cells three times in PBS buffer using a multichannel pipette with great care in order not to detach cells. 100 μl PBS was added to each well, cells were inspected in a light microscope and FFN206 uptake was quantified in a Tecan Spark plate reader using λ_ex_/λ_em_ of 369 nm/464 nm. Unspecific substrate uptake and background signal were measured in wells with Hek293 control cells and subtracted from the measured FFN206 signal in VMAT2-transfected Hek293 cells. For IC_50_ determination, compound concentrations ranging from 0.19 nM to 300 μM were used, and IC_50_ values were determined using a four-parameter dose-response curve in GraphPad Prism. Several VMAT2 mutants included in the mutagenesis analysis had no substrate transport and a reliable IC_50_ could thus not be calculated. VMAT2 Y434A had some substrate transport but no inhibition was observed by ZIP and TBZ within the range of the tested concentrations and IC_50_ values could thus not be calculated due to lack of inhibition, marked as ND (not determined) in Table 3.

### [^3^H]-DTBZ competition assay

Hek293 cells were grown and transfected with pcDNA3.1 *VMAT2*-His as described above. Two days after transfection, cells were harvested, washed and transferred to 96 well MultiScreen FB filter plates (Millipore). Cells where preincubated with hit compounds at 50 μM diluted in Opti-MEM for 30 min before [^3^H]-dihydrotetrabenazine (DTBZ; ViTrax Radiochemicals; 77 Ci/mmol) was added to a final concentration of 5 nM and incubated for 20 min at 37 °C. Cells were washed 3 times with ice cold PBS and 50 µl Ultima Gold LLL (PerkinElmer) was added to each well. [^3^H] scintillation counting was performed in a MicroBeta2 microplate counter (PerkinElmer). For EC_50_ determination, cells were preincubated for 30 min at 37 °C, with compound concentrations ranging from 100 μM to 1.7 nM before 5 nM [^3^H]-DTBZ was added to the cells. After a 20 min incubation at 37 °C, cells were washed and [^3^H] scintillation counting was performed as described above. EC_50_ values were determined using a four-parameter dose-response curve in GraphPad Prism.

To further investigate inhibitor binding, VMAT2-StrepII WT and VMAT2-StrepII mutants selected based on inhibitor binding modes identified by MD-simulations (VMAT2-StrepII Q143A, V233A-L234A, E313D, F335A-L336A, D400E, D400E-Y342H and Y434A mutants were expressed in Hek293 cells, bound to Strep-Tactin Sepharose beads (IBA Lifesciences GmbH) and assessed for [^3^H]-DTBZ and [^3^H]-salmeterol (SMT; ViTrax Radiochemicals; 11 Ci/mmol) binding. VMAT2 WT and mutants with the residue substitutions indicated in Fig. 6 were expressed in Hek293 cells, as described above in 6 or 10 cm dishes. Two days after transfection, cells were harvested in ice cold PBS and lyzed by sonication using a Sonics Vibra cell^TM^ sonicator and the insoluble fraction, including membranes, were collected by ultracentrifugation at 180,000 g for 1 h at 4 °C. The pellet was homogenized using a 1 ml dounce homogenizer, and membranes were solubilized in PBS buffer with 1% DDM / 0.1% CHS, and cOmplete ^TM^ EDTA free protease inhibitors for 1.5 h at 4 °C. Unsolubilized material was removed by centrifugation at 20,000 g for 30 min at 4 °C. VMAT2-Strep-tagII was purified by incubating the supernatant with Strep-Tactin Sepharose beads at 4 °C for 2 h. After incubation, beads were centrifuged for 2 min at 1000 g and resuspended and washed in PBS before being aliquoted and transferred to MultiScreen FB 96 well filter plates (Millipore). The Sepharose beads with VMAT2 were washed twice in PBS and incubated for 20 min either in 5 nM [^3^H]-DTBZ or 15 nM [^3^H]-SMT. Following incubation with radiolabeled inhibitors, plates were washed twice with PBS, and incubated with 50 µl Ultima Gold LLL. ^3^H scintillation counting was performed in a MicroBeta2 microplate counter. The amount of VMAT2 present in the samples was quantified by SDS-PAGE and western blotting using a StrepMAB-Classic HRP conjugate (IBA LifeSciences GmbH). Western blots were imaged using a Chemidoc XRS gel imaging system (Bio-Rad), and VMAT2-strepII bands were quantified using the Image Lab software from Bio-Rad. A representative western blot used for quantification are shown in Supplementary Fig. 4. Measured ^3^H inhibitor binding was then correlated to the amount of VMAT2 present in each sample and normalized to display the % of binding relative to inhibitor binding to VMAT2 WT.

### Molecular dynamics (MD) simulations

#### System setup

A detailed account of the preparation of ligand:VMAT2 complexes for MD simulations by means of molecular docking is provided in the Supplementary information (SI) section. For the modeling of the transporter we applied two rat VMAT2 homology models previously published^22,23^ and deposited to the Protein Model Database (PMDB)^59^; one cytoplasm-facing (VMAT2_CYT_; PMDB identifier PM0078823)^22^ and one lumen-facing (VMAT2_LUM_; PMDB identifier PM0080553)^23^. The PPM server^60^ (https://opm.phar.umich.edu/ppm_server) was applied in order to calculate optimal placements of apo-VMAT2_CYT_ and apo-VMAT2_LUM_ in membranes. Subsequently, the Charmm Membrane Builder^61,62^ used the PPM orientations to generate fully solvated membrane bilayer systems around the two models, which contained the following: 200 POPC lipids, 40 mol % cholesterol – corresponding to the cholesterol content in synaptic vesicle membranes^63^ – 19,920 water molecules, 150 mM NaCl and additional counterions to neutralize the net charge. Any lipids overlapping with, or too close to, the protein was removed.

Salmeterol (SMT) and ziprasidone (ZPS), the nanomolar inhibitors of VMAT2 identified in the screening, as well as reserpine (RSP) and tetrabenazine (TBZ), well-established VMAT2 inhibitors with yet unknown binding sites and binding modes, were selected as ligands for docking and MD simulation. The same ligand stereoisomers as used in the experiments were prepared for docking in Schrödinger Maestro (Schrödinger Release 2019-3: Maestro, Schrödinger, LLC, New York, NY, 2019) by means of the LigPrep module. All ligand states identified by LigPrep and given in Supplementary Table 2 were docked to both VMAT2_CYT_ and VMAT2_LUM_. A detailed account of the applied Glide and induced fit docking (IFD) procedures is provided in the SI. For each of the ligand states, the ligand:VMAT2_CYT_ and ligand:VMAT2_LUM_ complexes with the highest IFD score was aligned to and replaced the corresponding membrane-embedded apo-VMAT2. In the case of the positively charged ligands, one sodium ion was removed to maintain system neutrality. Antechamber^64^ assigned GAFF parameters^65^ and AM1-BCC charges^66^ to all the ligands. The ff14SB protein force field^67^ was applied to VMAT2 while maintaining the protonation states predicted by Maestro. The Lipid14 force field^68,69^ modeled POPC and cholesterol, ff99 ion parameters were used for NaCl^70^, and water molecules were of the TIP3P type^71^. Final system sizes mounted to 102,000–103,000 atoms.

#### Simulation details

All MD simulations were performed in periodic boundary conditions using the GPU-accelerated PMEMD engine^72,73^ in AMBER18^74^. The particle mesh Ewald (PME) method evaluated the electrostatic energies, and a 10 Å nonbonded cut-off was applied to the direct space sum and van der Waals interactions. Semi-isotropic pressure coupling with a reference value of 1 bar and a relaxation time of 1.0 ps was controlled by the Berendsen barostat, and the temperature regulated by means of the Langevin thermostat with a 1.0 ps^−1^ collision frequency. SHAKE allowed for 2 fs time steps by constraining the length of bonds involving hydrogen.

All ligand:VMAT2 systems were subjected to the following minimization and simulation stages: (1) 10,000-step minimization with restraints on VMAT2 and ligand heavy atoms; (2) 10,000-step minimization with restraints on VMAT2 backbone and ligand heavy atoms; (3) 10,000-step minimization without restraints; (4) 5 ps gradual heating from 0 to 100 K at constant volume with 10 kcal/mol·Å^2^ restraints on the ligand:VMAT2 complex; (5) 100 ps gradual heating from 100 to 310 K with 10 kcal/mol·Å^2^ restraints on the complex; (6) 45 ns equilibration at 310 K with gradual release of restraints on the VMAT2 backbone and ligand heavy atoms from 1 to 0 kcal/mol·Å^2^ (force constant halved every 5 ns) whilst keeping a very weak restraint on POPC phosphorus atoms; (7) Equilibration/simulation at 310 K with the complex fully released. Steps 5–7 were performed with semi-isotropic pressure coupling. Depending on the stability of the complex, the simulation time ranged from 500 ns to 1 µs in step 7. Each trajectory was RMSD fitted to the VMAT TM C-alpha atoms, before the RMSD of the ligand heavy atoms was calculated (Supplementary Figs. 5 and 6). We considered it to be a stable and plausible binding mode when the RMSD values of both the TM C-alpha atoms and the ligand had been stable for 200 ns, which was also the portion of the simulation used for analysis with CPPTRAJ^75^ and MMPBSA.py^76^. A clustering based on the position of the ligand in the already RMSD-fitted protein was performed in CPPTRAJ, and the representative structure of the stabilized complex was subsequently extracted as the centroid of the largest cluster. RMSD from ligand:VMAT2_CYT_ and ligand:VMAT2_LUM_ simulations are shown in Supplementary Figs. 5 and 6, respectively, and all the representative structures are presented in Supplementary Fig. 3, both as 3D representations of the whole complex and as 2D ligand interaction diagrams (LIDs). Based on their relevance to the discussion, some of the LIDs are also replicated in the main text (Fig. 5). The LIDs were generated by means of Schrödinger Maestro). Hydrogen bond occupancies calculated from the simulations using CPPTRAJ defaults have been added to the diagrams.

### MM/P(G)BSA calculations

When sufficiently stable, the MD simulations were post-processed using MM/P(G)BSA methods. We employed the single trajectory approach and extracted 1000 evenly spaced frames from the last 200 ns of each simulation that were subjected to the MM/P(G)BSA calculations outlined below using the AMBER18 MMPBSA.py interface^76^.

To approximate binding energies of the simulated ligand:VMAT2 complexes at reasonable computational cost we employed the single dielectric implicit membrane MM/PBSA method^77^ implemented in AMBER18^74^, where standard MM/PBSA has been extended to membrane protein systems. Solvation effects resulting from the lipid bilayer environment are accounted for by inclusion of an implicit membrane slab with a dedicated dielectric constant. In our MM/PBSA calculations, we set the protein dielectric constant to 2^78^ while the implicit membrane was assigned a dielectric constant of 4^77,78^, 34 Å thickness and a solvent probe of 2.7 Å. We set the water dielectric constant to 80, the water solvent probe to 1.4 Å and the ionic strength to 0.15 M. The nonpolar solvation energy was modeled as two separate terms; the dispersion term and the cavity term (corresponds to the inp = 2 setting in AMBER). All other relevant parameters were the same as in refs. ^77,78^. A drawback of our calculations is that the entropy term is omitted, implying that we are not computing binding free energy but rather binding enthalpy. This is however a fairly common MM/P(G)BSA approximation that was also applied in the paper describing the methodology^77^. Thus, in validating the method, Greene et. al analyzed binding of co-crystallized ligands to the human purinergic platelet receptor and found that calculated binding enthalpies correlated well with experimental binding affinities^77^. Evaluation of entropic contributions by normal mode analysis was considered too computationally demanding in light of the number of simulated ligand:VMAT complexes. Besides, entropy calculations tend to introduce additional noise and uncertainty without necessarily increasing the accuracy of binding energy predictions^79–82^.

Standard per-residue MM/GBSA energy decomposition was utilized for identifying the VMAT2 residues that contributed most favorably to the ligand binding enthalpy in each simulation. Polar solvation energies were estimated using the GB^HTC^ model with the parameters developed by Tsui and Case (igb = 1) and compatible mbondi atomic radii^83^. Salt concentration was set to 0.15 M. Surface area was determined by the LCPO method^84^ and a surface tension of 0.0072 kcal mol^−1^ Å^−2^ was used when calculating nonpolar solvation energies. Otherwise, default settings were applied.

### Statistics and reproducibility

DSF screening and DRA validation experiments are presented as mean ± SD of triplicates. IC_50_ values are presented as mean ± SD of best-fit values from ≥3 independent experiments with triplicates or quadruplicates unless otherwise stated in the figure legend. [^3^H]-inhibitor binding is reported as mean ± SD from three independent experiments with triplicates or quadruplicates unless otherwise stated in the figure legend. Statistical analysis was performed by one-way ANOVA tests as described in the figure legends, using GraphPad Prism 8.2.1. Data points influenced by experimental or technical errors was excluded from the analysis.

### Reporting summary

Further information on research design is available in the Nature Research Reporting Summary linked to this article.

## Supplementary information

Supplementary Information

Description of Additional Supplementary Files

Supplementary Data 1

NR reporting summary checklist

## Data Availability

Source data behind all graphs and figures can be found in the supplementary Data 1. Source data from docking and MD simulations are available from corresponding authors upon reasonable request.
