# Supplementary material for: Inhibition of VMAT2 by β2-adrenergic agonists, antagonists, and the atypical antipsychotic ziprasidone
Source: Commun Biol. 2022 Nov 23;5:1283. doi: 10.1038/s42003-022-04121-1 (PMC9684503; doi:10.1038/s42003-022-04121-1)
Supplement: Supplementary file 1 — Supplementary Information [file 42003_2022_4121_MOESM1_ESM.pdf]

SUPPLEMENTARY INFORMATION

Inhibition of VMAT2 by β2-adrenergic agonists, antagonists, and the atypical antipsychotic ziprasidone

Svein Isungset Støve[1,2#‡], Åge Aleksander Skjevik[1#], Knut Teigen[1], Aurora Martinez[1,2,3‡]

[1] Department of Biomedicine, University of Bergen, Jonas Lies vei 91, 5009 Bergen, Norway

[2]Neuro-SysMed, Department of Neurology, Haukeland University Hospital, 5021 Bergen, Norway

[3]K.G Jebsen Center for Translational Research in Parkinson's Disease, University of Bergen, 5020 Bergen, Norway

[#]Shared first authorship

[‡]Corresponding authors:

Svein Isungset Støve: Svein.stove@uib.no

Aurora Martinez: Aurora.martinez@uib.no

Table S1 All primary hits from the initial DSF screen with ΔT_m from DSF and IC_{50} values from substrate uptake assays. Hierarchial cluster number corresponds to Figure 1d. Primary hits that stabilize or destabilize VMAT2 are shown in green or red background, respectively. ΔTm values are the average change in melting temperature of VMAT2 compared to controls from a DSF assay with triplicates. IC50 values are the average IC50 value from 3 or more independent experiments, as determined by substrate uptake assays in transfected Hek293 cells.

Hierarchial Cluster	Structure	Library identifier	Chemical name	ΔT_m (°C)	IC_{50}, rat VMAT2	IC_{50}, human VMAT2	IC_{50}, human VMAT1	Class	Therapeutic effect	Previously known VMAT inhibitor	CAS_number	smiles_code
1	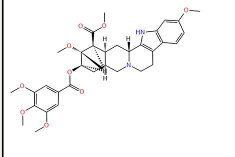	Prestw-875	Reserpine	13,73 ± 0,05				Central Nervous System	Antipsychotic	YES	50-55-5	c12[nH]c3c(c1CCN1[C@@]2(C[C@@]2([C@@H]([C@H]([C@H](OC(c4cc(c(c(c4)OC)OC)OC)=O)C[C@@]2(C1)[H])OC)C(=O)OC)[H])[H])ccc(c3)OC
2	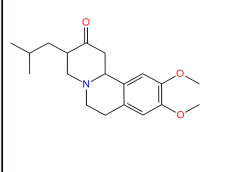	-	Tetrabenazine	8,17 ± 0,14	0,037 ± 0,028 µM	0,018 ± 0,004 µM	>100 µM	Central Nervous System	VMAT2 inhibitor	YES	58-46-8	COC1=C(OC)C=C2C3CC(=O)C(CC(C)C)CN3CCC2=C1
3	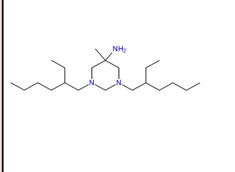	Prestw-551	Hexetidine	-3,96 ± 0,17				Infectiology	Antifungal	NO	141-94-6	N1(CN(CC(C1)(N)C)CC(CC)CCCC)CC(CC)CCCC
4	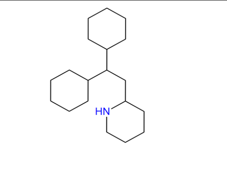	Prestw-286	Perhexiline maleate	-1,53 ± 0,17				Cardiovascular	Antianginal	NO	6724-53-4	C(CC1NCCCC1)(C1CCCCC1)C1CCCCC1
5	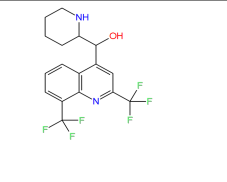	Prestw-126	Mefloquine hydrochloride	-11,15 ± 0,23				Infectiology	Antimalarial	NO	51773-92-3	c12nc(C(F)(F)F)cc(c1cccc2C(F)(F)F)C(C1NCCCC1)O
6	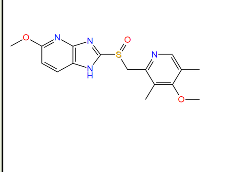	Prestw-1351	Tenatoprazole	7,36 ± 0,41	>10 µM			Metabolism	Antiulcer	NO	113712-98-4	n1c([nH]c2c1nc(cc2)OC)S(Cc1c(c(c(cn1)C)OC)C)=O
7	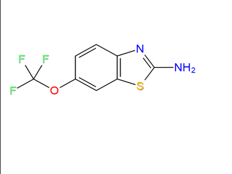	Prestw-167	Riluzole hydrochloride	-1,34 ± 0,22				Central Nervous System	Antispastic	NO	not available	c1(nc2c(s1)cc(OC(F)(F)F)cc2)N
8	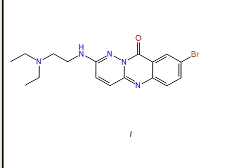	Prestw-1342	Sildenafil	1,10 ± 0,14				Cardiovascular	Antihypertensive	NO	139755-83-2	c1/2c(n(nc1CCC)C)C(N/C(=N2)/c1cc(S(N2CCN(CC2)C)(=O)=O)ccc1OCC)=O
9	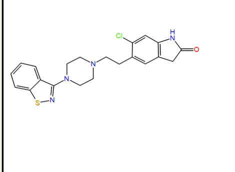	Prestw-1188	Ziprasidone Hydrochloride	2,69 ± 0,12	0,039 ± 0,017 µM	0,023 ± 0,018 µM	0,035 ± 0,017 µM	Central Nervous System	Antipsychotic	NO	138982-67-9	c1(nsc2c1cccc2)N1CCN(CCc2c(cc3NC(Cc3c2)=O)Cl)CC1
10	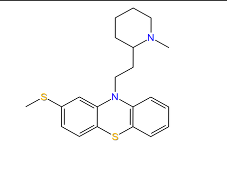	Prestw-78	Thioridazine hydrochloride	-6,13 ± 0,16				Central Nervous System	Antipsychotic	NO	130-61-0	N1(c2c(Sc3c1cccc3)ccc(c2)SC)CCC1N(C)CCCC1
10	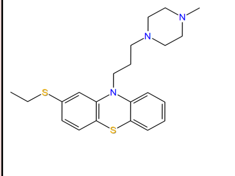	Prestw-1068	Thiethylperazine dimalate	-6,01 ± 0,16				Central Nervous System	Antiemetic	NO	52239-63-1	N1(c2c(Sc3c1cccc3)ccc(c2)SCC)CCCN1CCN(CC1)C
10	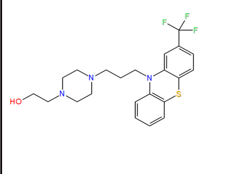	Prestw-320	Fluphenazine dihydrochloride	-5,24 ± 0,24				Central Nervous System	Antipsychotic	NO	146-56-5	N1(c2c(Sc3c1cccc3)ccc(C(F)(F)F)c2)CCCN1CCN(CC1)CCO
10	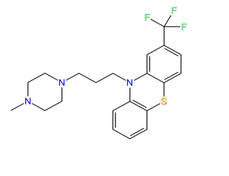	Prestw-313	Trifluoperazine dihydrochloride	-6,15 ± 0,08				Central Nervous System	Antiemetic	NO	440-17-5	N1(c2c(Sc3c1cccc3)ccc(C(F)(F)F)c2)CCCN1CCN(CC1)C

**10**	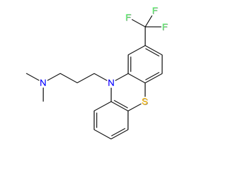	Prestw-53	Triflupromazine hydrochloride	-4,15 ± 0,03				Central Nervous System	Antiemetic	NO	1098-60-8	N1(c2c(Sc3c1cccc3)ccc(C(F)(F)F)c2)CCCN(C)C
**11**	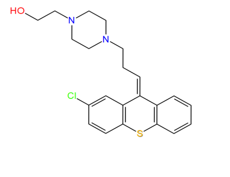	Prestw-998	Zuclopenthixol dihydrochloride	-1,95 ± 0,11				Central Nervous System	Antipsychotic	NO	633-59-0	C\1(/c2c(Sc3c1cccc3)ccc(c2)Cl)=C/CCN1CCN(CC1)CCO
**11**	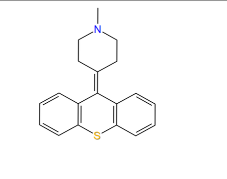	Prestw-294	Pimethixene maleate	-2,15 ± 0,27				Allergology	Antihistaminic	NO	13187-06-9	C\1(/c2c(Sc3c1cccc3)cccc2)=C\1/CCN(CC1)C
**11**	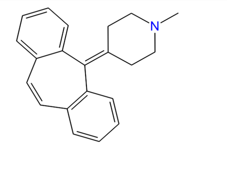	Prestw-103	Cyproheptadine hydrochloride	-1,43 ± 0,34				Allergology	Antihistaminic	NO	969-33-5	C\1(/c2c(\C=C/c3c1cccc3)cccc2)=C\1/CCN(CC1)C
**12**	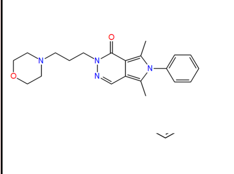	Prestw-368	Bepridil hydrochloride	-7,60 ± 0,07				Cardiovascular	Antianginal	NO	74764-40-2	N(CC(N1CCCC1)COCC(C)C)(Cc1ccccc1)c1ccccc1
**13**	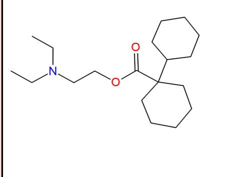	Prestw-48	Dicyclomine hydrochloride	-0,82 ± 0,06				Gastroenterology	Antispastic	NO	67-92-5	C1(C(=O)OCCN(CC)CC)(C2CCCCC2)CCCCC1
**14**	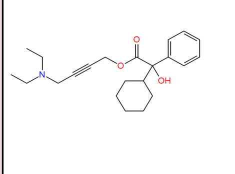	Prestw-287	Oxybutynin chloride	-12,41 ± 0,71				Neuromuscular	Antispastic	NO	1508-65-2	C(C(=O)OCC#CCN(CC)CC)(c1ccccc1)(C1CCCCC1)O
**14**	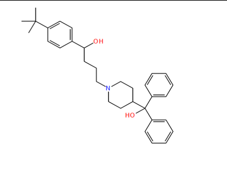	Prestw-138	Terfenadine	-13,22 ± 0,13				Allergology	Antihistaminic	NO	50679-08-8	C(C1CCN(CC1)CCCC(c1ccc(C(C)(C)C)cc1)O)(c1ccccc1)(c1ccccc1)O
**14**	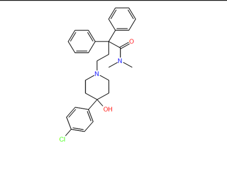	Prestw-144	Loperamide hydrochloride	-7,10 ± 0,28	1,930 ± 1,301 µM			Gastroenterology	Antidiarrheal	NO	34552-83-5	C(C(CCN1CCC(CC1)(c1ccc(cc1)Cl)O)(c1ccccc1)c1ccccc1)(N(C)C)=O
**14**	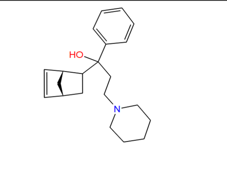	Prestw-502	Biperiden hydrochloride	1,32 ± 0,22				Central Nervous System	Antiparkinsonian	NO	1235-82-1	C(C1[C@]2(\C=C/[C@@](C1)(C2)[H])[H])(CCN1CCCCC1)(c1ccccc1)O.Cl
**15**	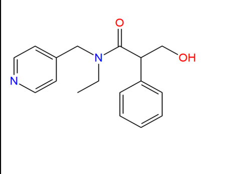	Prestw-228	Tropicamide	0,80 ± 0,08				Neuromuscular	Mydriatic	NO	1508-75-4	C(C(c1ccccc1)CO)(N(Cc1ccncc1)CC)=O
**15**	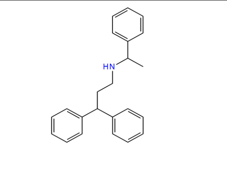	Prestw-270	Fendiline hydrochloride	-4,45 ± 0,12				Cardiovascular	Antianginal	NO	13636-18-5	C(c1ccccc1)(c1ccccc1)CCNC(c1ccccc1)C
**15**	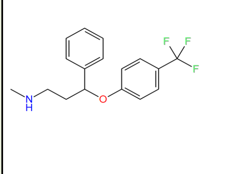	Prestw-511	Fluoxetine hydrochloride	1,55 ± 0,14	1,985 ± 0,254 µM			Central Nervous System	Antidepressant	YES	59333-67-4	C(c1ccc(OC(c2ccccc2)CCNC)cc1)(F)(F)F
**16**	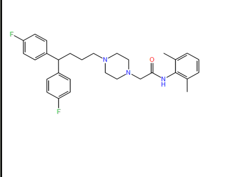	Prestw-381	Lidoflazine	1,35 ± 0,18				Cardiovascular	Antianginal	NO	3416-26-0	c1(NC(CN2CCN(CC2)CCCC(c2ccc(cc2)F)c2ccc(cc2)F)=O)c(cccc1C)C
**16**	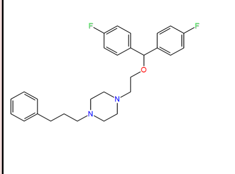	Prestw-386	GBR 12909 dihydrochloride	-17,11 ± 0,01				Central Nervous System	Antidepressant	YES	67469-78-7	N1(CCN(CC1)CCCc1ccccc1)CCOC(c1ccc(cc1)F)c1ccc(cc1)F
**16**	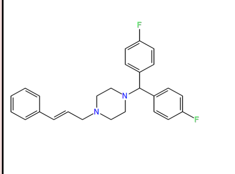	Prestw-312	Flunarizine dihydrochloride	-6,11 ± 0,34				Central Nervous System	Anticonvulsant	NO	30484-77-6	N1(C(c2ccc(cc2)F)c2ccc(cc2)F)CCN(CC1)C/C=C/c1ccccc1

**16**	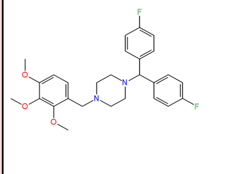	Prestw-1775	Lomerizine hydrochloride	-7,51 ± 0,04	3,898 ± 1,688 µM			Central Nervous System	Antimigraine	NO	101477-54-7	c1(c(c(CN2CCN(C(c3ccc(cc3)F)c3ccc(cc3)F)CC2)ccc1OC)OC)OC
**17**	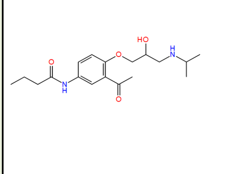	Prestw-69	Acebutolol hydrochloride	2,14 ± 0,06	<10 µM			Cardiovascular	Antianginal	NO	34381-68-5	c1(c(OCC(CNC(C)C)O)ccc(NC(=O)CCC)c1)C(=O)C
**17**	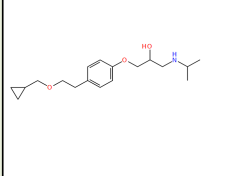	Prestw-382	Betaxolol hydrochloride	1,98 ± 0,15	7.337 ± 1.370 µM			Cardiovascular	Antiglaucoma	NO	63659-19-8	C1CC1COCCc1ccc(OCC(CNC(C)C)O)cc1
**17**	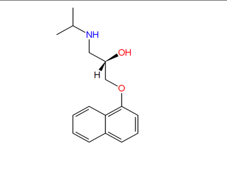	Prestw-1075	(R)-Propranolol hydrochloride	2,63 ± 0,13	2.211 ± 0.156 µM			Cardiovascular	Antianginal	NO	13071-11-9	c12c(OCC(CNC(C)C)(O)[H])cccc1cccc2
**17**	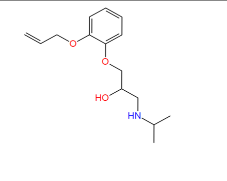	Prestw-1057	Oxprenolol hydrochloride	2,51 ± 0,08	6.548 ± 3.494 µM			Cardiovascular	Antianginal	NO	6452-73-9	O(c1c(OCC=C)cccc1)CC(CNC(C)C)O
**17**	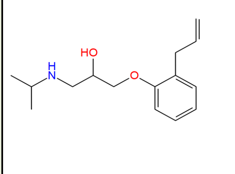	Prestw-250	Alprenolol hydrochloride	2,30 ± 0,05				Cardiovascular	Antianginal	NO	13707-88-5	O(c1c(CC=C)cccc1)CC(CNC(C)C)O
**17**	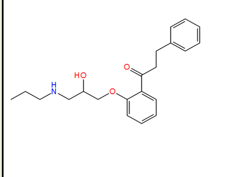	Prestw-499	Propafenone hydrochloride	4,53 ± 0,18	3.384 ± 1.865 µM			Cardiovascular	Antiarrhythmic	NO	34183-22-7	c1(C(=O)CCc2ccccc2)c(OCC(O)CNCCC)cccc1
**18**	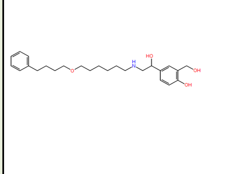	Prestw-945	Salmeterol	9,71 ± 0,22	0.053 ± 0.028 µM	0.035 ± 0.016 µM	0.023 ± 0.002 µM	Respiratory	Bronchodilator	NO	89365-50-4	c1(cc(ccc1O)C(O)CNCCCCCCOCCCCc1ccccc1)CO
**18**	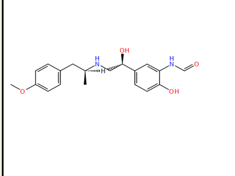	Prestw-1421	Formoterol fumarate	1,87 ± 0,19	0.565 ± 0.314 µM			Respiratory	Antiasthmatic	NO	43229-80-7	c1(cc(ccc1O)[C@@H](CN[C@H](Cc1ccc(cc1)OC)C)O)NC=O.c1(cc(ccc1O)[C@@H](CN[C@H](Cc1ccc(cc1)OC)C)O)NC=O
**18**	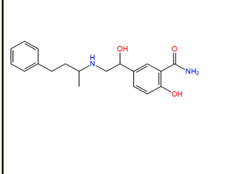	Prestw-277	Labetalol hydrochloride	0,81 ± 0,07				Respiratory	Antiasthmatic	NO	32780-64-6	c1(cc(ccc1O)C(CNC(CCc1ccccc1)C)O)C(=O)N
**18**	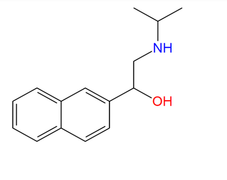	Prestw-974	Pronethalol hydrochloride	2,42 ± 1,26	1.011 ± 0.300 µM			Cardiovascular	Antianginal	NO	51-02-5	c1(cc2c(cc1)cccc2)C(CNC(C)C)O
**19**	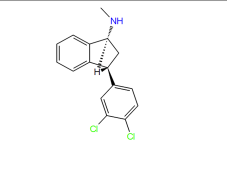	Prestw-1602	Indatraline hydrochloride	-4,93 ± 0,09				Central Nervous System	Antidepressant	NO	86939-10-8	[C@@H]1(c2c([C@@H](C1)NC)cccc2)c1cc(c(cc1)Cl)Cl
**20**	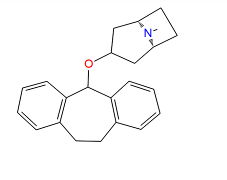	Prestw-1013	Deptropine citrate	-3,11 ± 0,03				Allergology	Antihistaminic	NO	2169-75-7	C1(c2c(CCc3c1cccc3)cccc2)OC1C[C@@H]2N([C@H](C1)CC2)C
**21**	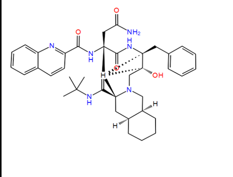	Prestw-1114	Saquinavir mesylate	-4,29 ± 0,17				Immunology	Antiviral	NO	149845-06-7	N1([C@H](C(NC(C)(C)C)=O)C[C@]2([C@@](C1)(CCCC2)[H])[H])C[C@H]([C@@H](NC([C@@H](NC(c1nc2c(cc1)cccc2)=O)CC(=O)N)=O)Cc1ccccc1)O
**22**	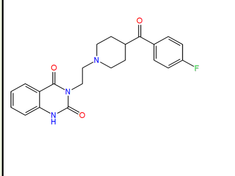	Prestw-392	Ketanserin tartrate hydrate	3,12 ± 0,24				Cardiovascular	Antihypertensive	YES	83846-83-7	N1(C(Nc2c(C1=O)cccc2)=O)CCN1CCC(C(c2ccc(cc2)F)=O)CC1
**23**	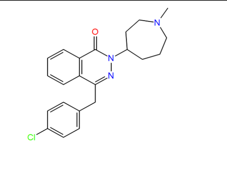	Prestw-1130	Azelastine HCl	0,85 ± 0,24				Immunology	Antihistaminic	NO	79307-93-0	N1(/N=C(\c2c(C1=O)cccc2)/Cc1ccc(Cl)cc1)C1CCN(CCC1)C

**24**	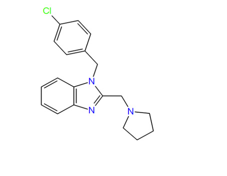	Prestw-227	Clemizole hydrochloride	-1,46 ± 0,10				Allergology	Antihistaminic	NO	1163-36-6	c1(n(c2c(n1)cccc2)Cc1ccc(Cl)cc1)CN1CCCC1
**24**	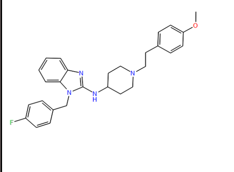	Prestw-136	Astemizole	-7,04 ± 0,40	1.171 ± 0.573 µM			Allergology	Antihistaminic	NO	68844-77-9	c1(n(c2c(n1)cccc2)Cc1ccc(F)cc1)NC1CCN(CC1)CCc1ccc(cc1)OC
**25**	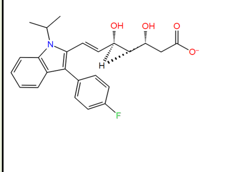	Prestw-859	Fluvastatin sodium salt	4,98 ± 0,17				Cardiovascular	Antilipemic	NO	93957-55-2	c1(n(c2c(c1c1ccc(cc1)F)cccc2)C(C)C)/C=C/[C@@H](C[C@@H](CC([O-])=O)O)O
**26**	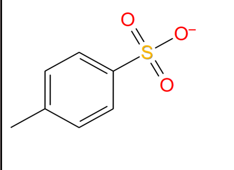	Prestw-319	Clofilium tosylate	-3,80 ± 0,20				Cardiovascular	Antiarrhythmic	NO	92953-10-1	[N+](CC)(CC)(CCCCCCC)CCCCc1ccc(Cl)cc1
**27**	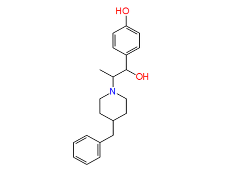	Prestw-311	Ifenprodil tartrate	1,23 ± 0,40				Cardiovascular	Vasodilator	NO	23210-58-4	N1(C(C(c2ccc(cc2)O)O)C)CCC(Cc2ccccc2)CC1
**28**	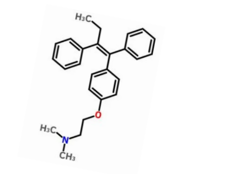	Prestw-146	Tamoxifen citrate	-5,92 ± 0,47				Endocrinology	Antineoplastic	NO	54965-24-1	C(=C(\c1ccccc1)/CC)(\c1ccc(cc1)OCCN(C)C)/c1ccccc1
**29**	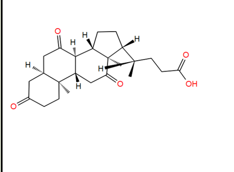	Prestw-123	Dehydrocholic acid	1,51 ± 0,28				Gastroenterology	Choleretic	NO	81-23-2	[C@]12([C@]([C@]3([C@@]([C@@]4([C@](CC3=O)(CC(=O)CC4)[H])C)(CC1=O)[H])[H])(CC[C@@H]2[C@@H](CCC(=O)O)C)[H])C
**29**	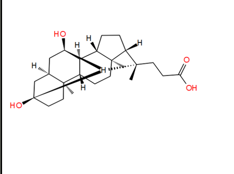	Prestw-285	Chenodiol	1,98 ± 0,15				Gastroenterology	Cholagogue	NO	474-25-9	[C@]12([C@]([C@]3([C@@]([C@@]4([C@](C[C@H]3O)(C[C@@H](CC4)O)[H])C)(CC1)[H])[H])(CC[C@@H]2[C@@H](CCC(=O)O)C)[H])C
**30**	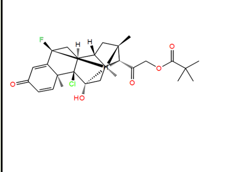	Prestw-1119	Clocortolone pivalate	1,04 ± 0,11				Endocrinology	Anti-inflammatory	NO	34097-16-0	[C@]\1\2([C@@]3([C@]([C@]4([C@@]([C@H]([C@@H](C4)C)C(COC(C(C)(C)C)=O)=O)(C[C@@H]3O)C)[H])(C[C@@H](/C1=C/C(\C=C2)=O)F)[H])Cl)C

Table S2 Validation of primary hits by dose response assays. Purified rVMAT2-His was mixed with CPM and compounds at increasing concentrations, and the protein melting temperature was determined by DSF and compared with the protein melting temperature of the DMSO controls. Experiments were performed in triplicates and the ΔT_m values are reported as the average ΔT_m ± standard deviation (SD)

Prestwick ID	Compound Name		Compound (µM)							
			270	90	30	10	3,33	1,11	0,37	0,12
**Medium-high affinity hits (validated compounds)**										
Prestw-945	Salmeterol	ΔT_m (°C)	10,46	8,70	7,90	7,48	7,04	6,55	6,25	5,78
		SD	0,28	0,22	0,27	0,15	0,41	0,47	0,30	0,28
Prestw-1351	Tenatoprazole	ΔT_m (°C)	6,79	6,56	6,25	4,77	4,85	1,30	0,24	0,38
		SD	0,02	0,18	0,36	1,39	1,37	0,17	0,14	0,25
Positive control	Tetrabenazine	ΔT_m (°C)	5,17	4,64	4,27	6,86	5,92	5,52	5,05	4,89
		SD	3,68	3,36	2,48	0,54	0,79	0,69	0,68	0,93
Prestw-499	Propafenone hydrochloride	ΔT_m (°C)	4,45	3,49	2,63	1,65	1,22	1,22	0,99	1,09
		SD	0,46	0,05	0,24	0,52	0,38	0,39	0,00	0,14
Prestw-511	Fluoxetine hydrochloride	ΔT_m (°C)	4,21	3,22	2,73	2,37	1,92	1,58	1,51	1,33
		SD	0,07	0,07	0,10	0,27	0,08	0,13	0,29	0,29
Prestw-974	Pronethalol hydrochloride	ΔT_m (°C)	3,75	2,42	1,83	0,71	0,48	0,70	0,21	-0,02
		SD	1,33	1,26	0,72	0,00	0,16	0,60	0,26	0,25
Prestw-859	Fluvastatin sodium salt	ΔT_m (°C)	2,74	1,39	1,05	0,87	0,66	0,71	0,66	0,62
		SD	0,12	0,11	0,15	0,03	0,09	0,04	0,10	0,18
Prestw-1057	Oxprenolol hydrochloride	ΔT_m (°C)	2,47	0,89	0,78	0,41	0,14	0,00	0,06	0,04
		SD	0,62	0,12	0,30	0,00	0,00	0,07	0,02	0,25
Prestw-1421	Formoterol fumarate	ΔT_m (°C)	2,29	1,16	0,92	0,79	0,70	0,60	0,43	0,33
		SD	0,63	0,56	0,36	0,51	0,38	0,43	0,39	0,49
Prestw-1075	(R)-Propranolol hydrochloride	ΔT_m (°C)	2,25	1,45	1,12	1,04	0,89	0,58	0,73	0,71
		SD	1,11	0,70	0,69	0,51	0,44	0,43	0,46	0,29
Prestw-392	Ketanserin tartrate hydrate	ΔT_m (°C)	2,18	0,95	1,17	1,16				
		SD	0,24	0,51	0,25	0,71				
Prestw-502	Biperiden hydrochloride	ΔT_m (°C)	2,12	0,90	1,60	1,11	0,56	0,45	0,33	0,20
		SD	0,51	0,11	0,75	0,00	0,33	0,11	0,32	0,08
Prestw-1188	Ziprasidone Hydrochloride	ΔT_m (°C)	1,82	1,13	0,71	0,60	0,58	0,69	0,68	0,41
		SD	0,62	0,23	0,16	0,22	0,09	0,10	0,05	0,29
Prestw-123	Dehydrocholic acid	ΔT_m (°C)	1,43	0,53	0,66	0,39				
		SD	0,29	0,35	0,08	0,04				
Prestw-386	GBR 12909 dihydrochloride	ΔT_m (°C)	-5,40	-2,30	-1,67	-1,07	-0,34	0,05	-0,01	-0,02
		SD	0,54	0,20	0,20	0,16	0,21	0,18	0,20	0,17
Prestw-1775	Lomerizine hydrochloride	ΔT_m (°C)	-5,36	-2,04	-1,43	-2,21	-1,47	-1,46	-0,99	-0,81
		SD	2,17	0,94	0,47	0,24	0,84	0,76	0,49	0,34
Prestw-144	Loperamide hydrochloride	ΔT_m (°C)	-4,37	-2,50	-2,15	-1,75	-1,47	-1,24	-1,00	-0,88
		SD	0,10	0,19	0,14	0,10	0,07	0,14	0,15	0,05
Prestw-136	Astemizole	ΔT_m (°C)	-4,06	-2,06	-1,75	-1,56	-1,40	-0,80	-0,76	-0,38
		SD	0,32	0,33	0,30	0,58	0,64	0,35	0,33	0,35
Prestw-368	Bepridil hydrochloride	ΔT_m (°C)	-3,80	-1,63	-1,20	-1,08	-0,93	-0,75	-0,68	-0,78
		SD	0,07	0,01	0,21	0,06	0,04	0,11	0,09	0,12
Prestw-1068	Thiethylperazine dimalate	ΔTm	-3,69	-2,15	-1,34	-1,01	-0,78	-0,77	-0,58	-0,61
		SD	1,60	0,27	0,73	0,50	0,29	0,49	0,19	0,11
Prestw-312	Flunarizine dihydrochloride	ΔT_m (°C)	-3,51	-1,62	-1,21	-0,99	-0,43	-0,85	-0,65	-0,41
		SD	0,18	0,24	0,19	0,08	0,28	0,09	0,20	0,09

Prestw-270	Fendiline hydrochloride	ΔT_m (°C)	-3,27	-1,16	-0,24	-0,22				
		SD	1,43	0,92	0,51	0,19				
Prestw-286	Perhexiline maleate	ΔT_m (°C)	-3,23	-1,17	-0,27	-0,18				
		SD	1,36	0,06	0,13	0,00				
Prestw-146	Tamoxifen citrate	ΔT_m (°C)	-2,95	-1,22	-0,91	-0,83	-0,69	-0,40	-0,23	-0,29
		SD	0,39	0,40	0,31	0,33	0,19	0,29	0,28	0,38
Prestw-78	Thioridazine hydrochloride	ΔT_m (°C)	-2,90	-1,51	-1,19	-0,82	-0,72	-0,55	-0,37	-0,75
		SD	0,15	0,15	0,27	0,15	0,07	0,18	0,08	0,19
Prestw-319	Clofilium tosylate	ΔT_m (°C)	-2,26	-0,36	-0,15	-0,06				
		SD	0,72	0,34	0,16	0,29				
Prestw-1602	Indatraline hydrochloride	ΔT_m (°C)	-2,11	-1,01	-0,63	-0,37	-0,41	-0,28	0,04	0,24
		SD	0,58	0,41	0,31	0,22	0,40	0,15	0,30	0,12
Prestw-320	Fluphenazine dihydrochloride	ΔT_m (°C)	-1,86	-1,46	-0,55	-0,37	-0,44	-0,36	-0,08	
		SD	0,05	0,00	0,14	0,08	0,10	0,11	0,02	
Prestw-1114	Saquinavir mesylate	ΔT_m (°C)	-1,64	-0,55	-0,70	-0,91	-0,65	-0,49	-0,28	-0,17
		SD	1,49	0,43	0,58	0,83	0,20	0,31	0,20	0,22
Prestw-998	Zuclopenthixol dihydrochloride	ΔT_m (°C)	-1,51	-0,79	-1,80	-0,07	0,03	-0,58	-0,44	-0,36
		SD	0,45	0,35	0,00	0,51	0,33	0,25	0,31	0,23
Prestw-875	Reserpine	ΔT_m (°C)	13,73	13,99	13,92	13,52	13,60	13,61	13,66	13,68
		SD	0,04	0,09	0,06	0,25	0,50	0,20	0,23	0,00
**Medium-low affinity or non-validated primary hits**										
Prestw-285	Chenodiol	ΔT_m (°C)	2,24	0,57	0,35	0,64	0,19	0,17	-0,01	-0,11
		SD	0,69	0,54	0,20	0,74	0,54	0,36	0,22	0,11
Prestw-250	Alprenolol hydrochloride	ΔT_m (°C)	1,21	0,40	0,73	0,14	0,02	0,04	-0,04	-0,11
		SD	0,12	0,05	0,61	0,05	0,02	0,06	0,07	0,11
Prestw-382	Betaxolol hydrochloride	ΔT_m (°C)	1,16	0,20	0,01	0,12	0,01	0,01	-0,34	-0,32
		SD	0,19	0,09	0,04	0,10	0,11	0,29	0,15	0,02
Prestw-381	Lidoflazine	ΔT_m (°C)	1,14	0,33	0,21	0,23	0,16	0,12	0,05	0,23
		SD	0,16	0,13	0,11	0,10	0,12	0,15	0,15	0,23
Prestw-1342	Sildenafil	ΔT_m (°C)	1,04	0,39	0,10	0,11	0,09	0,04	0,04	0,43
		SD	0,86	0,49	0,34	0,32	0,18	0,20	0,22	0,32
Prestw-69	Acebutolol hydrochloride	ΔT_m (°C)	0,90	0,33	0,31	0,20	0,03	0,30	0,03	0,20
		SD	0,28	0,19	0,19	0,04	0,13	0,50	0,28	0,29
Prestw-1130	Azelastine	ΔT_m (°C)	0,55	0,23	0,35	-0,60				
		SD	0,41	0,34	0,23	0,51				
Prestw-311	Ifenprodil tartrate	ΔT_m (°C)	0,41	-0,04	0,06	0,09	0,14	-0,20	0,32	0,30
		SD	0,13	0,12	0,20	0,31	0,36	0,12	0,39	0,67
Prestw-552	Selegiline hydrochloride	ΔT_m (°C)	0,36	0,28	0,19	0,30	0,32	0,31	0,38	0,33
		SD	0,18	0,14	0,09	0,13	0,04	0,07	0,09	0,02
Prestw-228	Tropicamide	ΔT_m (°C)	0,25	0,48	0,53	0,31				
		SD	0,24	0,24	0,32	0,10				
Prestw-277	Labetalol hydrochloride	ΔT_m (°C)	0,24	0,10	0,42	0,27				
		SD	0,13	0,19	0,10	0,05				
Prestw-126	Mefloquine hydrochloride	ΔT_m (°C)	0,08	0,09	0,21	0,10	-0,11	0,00	0,04	-0,02
		SD	0,07	0,09	0,04	0,23	0,13	0,11	0,12	0,19
Prestw-53	Triflupromazine hydrochloride	ΔT_m (°C)	-2,21	-0,84	-0,10	-0,10				
		SD	0,75	0,10	0,05	0,09				
Prestw-294	Pimethixene maleate	ΔT_m (°C)	-1,59	-0,14	-0,18	0,15				
		SD	0,25	0,41	0,19	0,18				

Prestw-551	Hexetidine	ΔT_m (°C)	-1,54	-0,46	-0,30	-0,28	-0,07	-0,02	-0,06	-0,09
		SD	0,33	0,24	0,24	0,21	0,06	0,09	0,04	0,21
Prestw-287	Oxybutynin chloride	ΔT_m (°C)	-1,54	-0,49	0,09	-0,39	-0,22	-0,15	-0,14	0,11
		SD	0,97	0,15	0,50	0,19	0,33	0,19	0,21	0,28
Prestw-103	Cyproheptadine hydrochloride	ΔT_m (°C)	-1,20	-0,41	-0,09	0,19				
		SD	0,22	0,04	0,02	0,04				
Prestw-313	Trifluoperazine dihydrochloride	ΔT_m (°C)	-1,05	-0,23	-0,28	-0,69				
		SD	0,27	0,72	0,41	0,31				
Prestw-227	Clemizole	ΔT_m (°C)	-0,69	0,31	0,57	0,33				
		SD	0,20	0,41	0,40	0,38				
Prestw-138	Terfenadine	ΔT_m (°C)	-0,60	-0,01	0,24	0,21				
		SD	0,31	0,08	0,11	0,04				
Prestw-167	Riluzole hydrochloride	ΔT_m (°C)	-0,54	0,30	0,38	0,31				
		SD	0,12	0,13	0,20	0,01				

Supplementary Table 3. Ligand states featured in the molecular modelling and the corresponding denotations used in text and figures. From the same stereoisomers as used in the experiments, the Schrödinger Maestro LigPrep module (Schrödinger Release 2019-3: Maestro, Schrödinger, LLC, New York, NY, 2019) generated the below ligand states. The red asterisk labels the identified protonation site in each ligand. Protonation of the relevant nitrogen in reserpine and tetrabenazine gives rise to another chiral center and to the protonated R and S forms predicted by LigPrep. All the ligand states were docked to each of the two VMAT states and the resulting complexes subjected to MD simulation and MM/P(G)BSA calculations.

Ligand		Ligand state	Denotation
**Reserpine (RSP)**	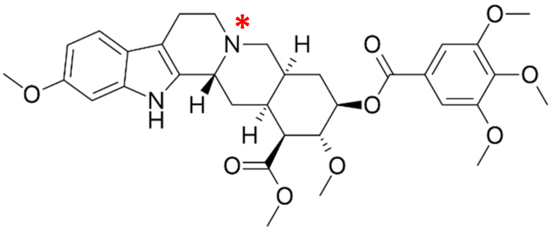	Deprotonated	RSP$_{deprot}$
		Protonated R	(R)-RSP+
		Protonated S	(S)-RSP+
**Tetrabenazine (TBZ)**	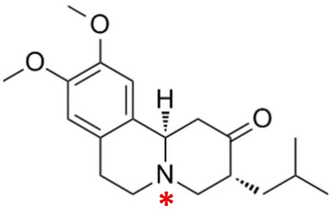	Deprotonated	TBZ$_{deprot}$
		Protonated R	(R)-TBZ+
		Protonated S	(S)-TBZ+
**Salmeterol (SMT)**	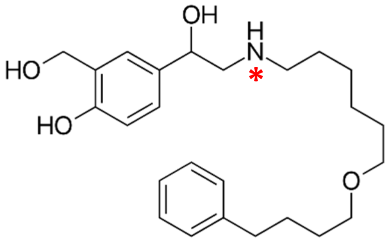	Protonated R	(R)-SMT+
		Protonated S	(S)-SMT+
**Ziprasidone (ZPS)**	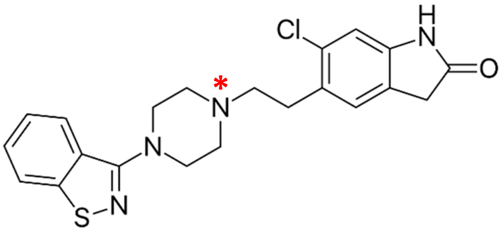	Deprotonated	ZPS$_{deprot}$
		Protonated	ZPS+

Supplementary Table 4. Previous studies of rat VMAT2 variants.

	[^{3}H]-serotonin uptake	[^{3}H]-tetrabenazine binding	Reference
**V132**	V132A: > 10x increased K_M<br>V132G: >5x increased K_M	V132A: >10x increase K_D<br>V132G: no TBZ binding	[1]
**G133**	G133A: No detectable activity<br>G133L: No detectable activity<br>G133P: No detectable activity	G133A: 50% reduction in binding<br>G133L: >90% reduction<br>G133P: >90% reduction	[1]
**F136**	F136L: >60% reduction of V_{max}	F136L: >45 x increased IC50	[2]
**K139**	K139A: >70% reduction<br>K139Y: > 95% reduction	K139A: 4.5x increased K_D<br>K139Y: 5x increased K_D	[3]
**K139 + D427**	K139A-D427A: 85% reduction<br>K139Y-D427N: 95% reduction	K139A-D427A: 7x increased K_D	[3]
**K139 + Q143 + D429**	K139A-Q143A-D427A: >95% reduction<br>K139A-Q143E-D427N: >70% reduction	K139A-Q143A-D427A: 16x increased K_D<br>K139A-Q143E-D427A: 6x increased K_D	[3]
**Q143**	Q143A: >40% decrease<br>Q143E: >40% increase<br>Q143N: >40% decrease	Q143A: 1.5 x increased K_D<br>Q143E: similar K_D<br>Q143N: similar K_D	[3]
**Q143 + D427**	Q143E-D427N: >80% reduction	Q143A-D427A: 1.5x increased K_D<br>Q143A-D427N: 7x increased K_D	[3]
**V233 + L234**	V233A-L234A: >95% reduction	V233A-L234A: 1.5x increased K_D	[3]
**V233 + L234 + F335 + L336**	V233A-L234A-F335A-L336A: >90% reduction	V233A-L234A-F335A-L336A: > 20x K_D	[3]
**E313**	E313D: 60% reduction<br>E313Q: 90% reduction	E313D: no binding<br>E313Q: no binding	[3, 4]
**P314**	P314L: >5x increased K_M<br>P314T: >10x increased K_M<br>P314G: >5x increased K_M	P314L: >20x increase K_D<br>P314T: >20x increase K_D<br>P314G: >20x increase K_D	[1]
**A315**	A315T: >60% reduction	A315T: 2x increase IC_{50}	[5]
**Y342**	Y342H: No effect<br>Y342S: >90% reduction<br>Y342F: >90% reduction<br>Y342Q: >90% reduction	Y342H: 50% lovered K_D	[3]
**Y342 + D400**	Y342H-D400E: >95% reduction<br>Y342Q-D400E: >95% reduction<br>Y342S:D400E: >95% reduction	Y342H + D400: 8x increase K_D	[3]
**F335 + L336**	F335A-L336A: >95% reduction	F335A-L336A: 8x increased K_D	[3]
**D400**	D400E 50% reduction<br>D400S: >95% reduction<br>D400C: >95% reduction	D400E: 3x increase K_D<br>D400S: 2x increase K_D	[3]
**D427**	D427A: >95% reduction<br>D427N: >90% reduction<br>D427E: >75% reduction	D427E: 3x increased K_D<br>D427N: 4.5x increased K_D<br>D427A: 4x increased K_D	[3]
**Y434**	Y434A: Slightly higher affinity (lower K_m)<br>Y434F: Slightly higher affinity (lower K_m)	Y434A: 25x increased IC_{50}<br>Y434F: 2x reduced IC_{50}	[5]

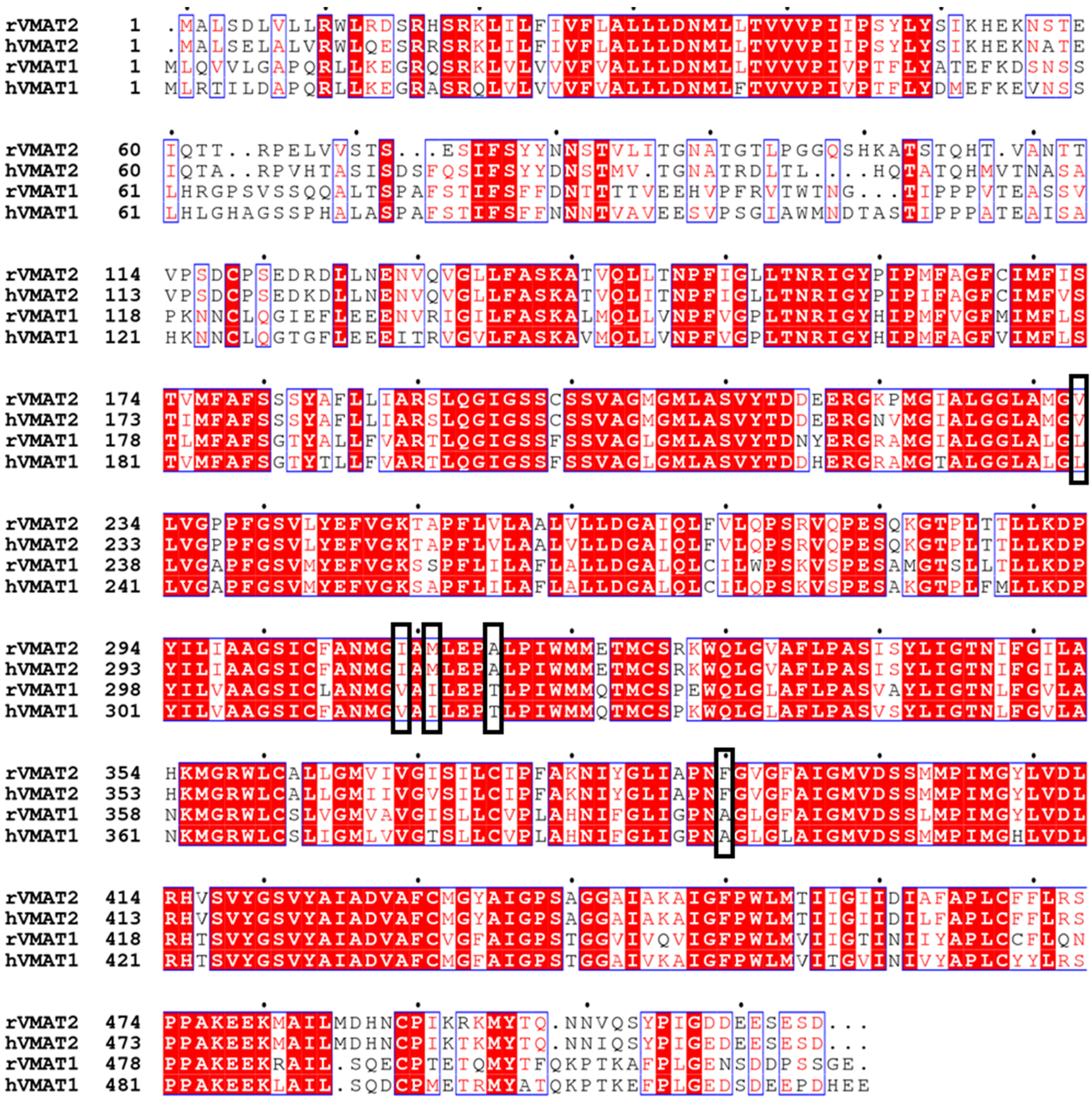

Supplementary Figure 1. Sequence alignment. Alignment of the rat (r) and human (h) VMAT2 and VMAT1 isoforms. The black boxes highlight main contributors to favourable binding enthalpy among the TBZ simulation systems that are identical in rVMAT2 and hVMAT2 but replaced by a different residue in their VMAT1 counterparts. The alignment was performed using Clustal Omega [6] (https://www.ebi.ac.uk/Tools/msa/clustalo/) and the resulting figure produced using ESPript [7] (https://espript.ibcp.fr). hVMAT2 and rVMAT2 have 91% sequence identity (94% positives in the BLOSUM62 scoring matrix). hVMAT1 and hVMAT2 have a 65% sequence identity (79% positives).

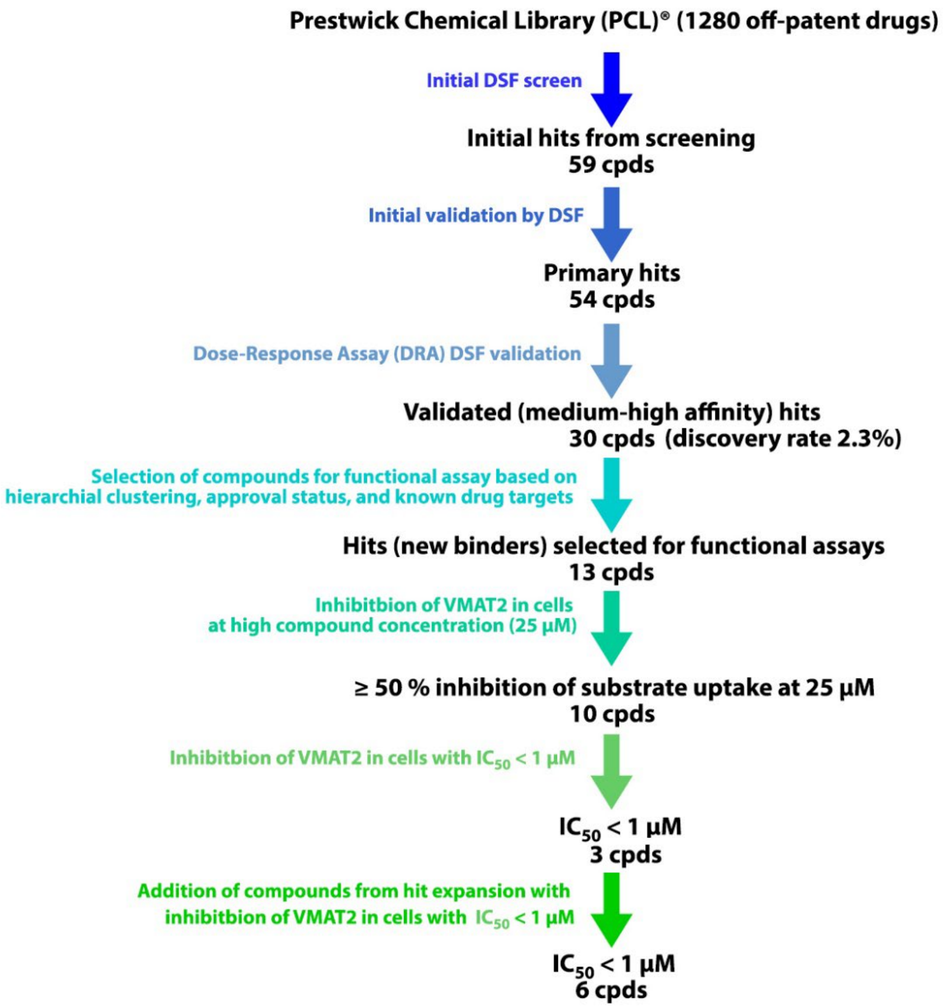

Supplementary Figure 2. Outline of all steps from the primary hit compounds to hit compounds selected for functional assays, to the final newly identified VMAT2 inhibitors with IC_{50} <1 μM. Cpd, compound.

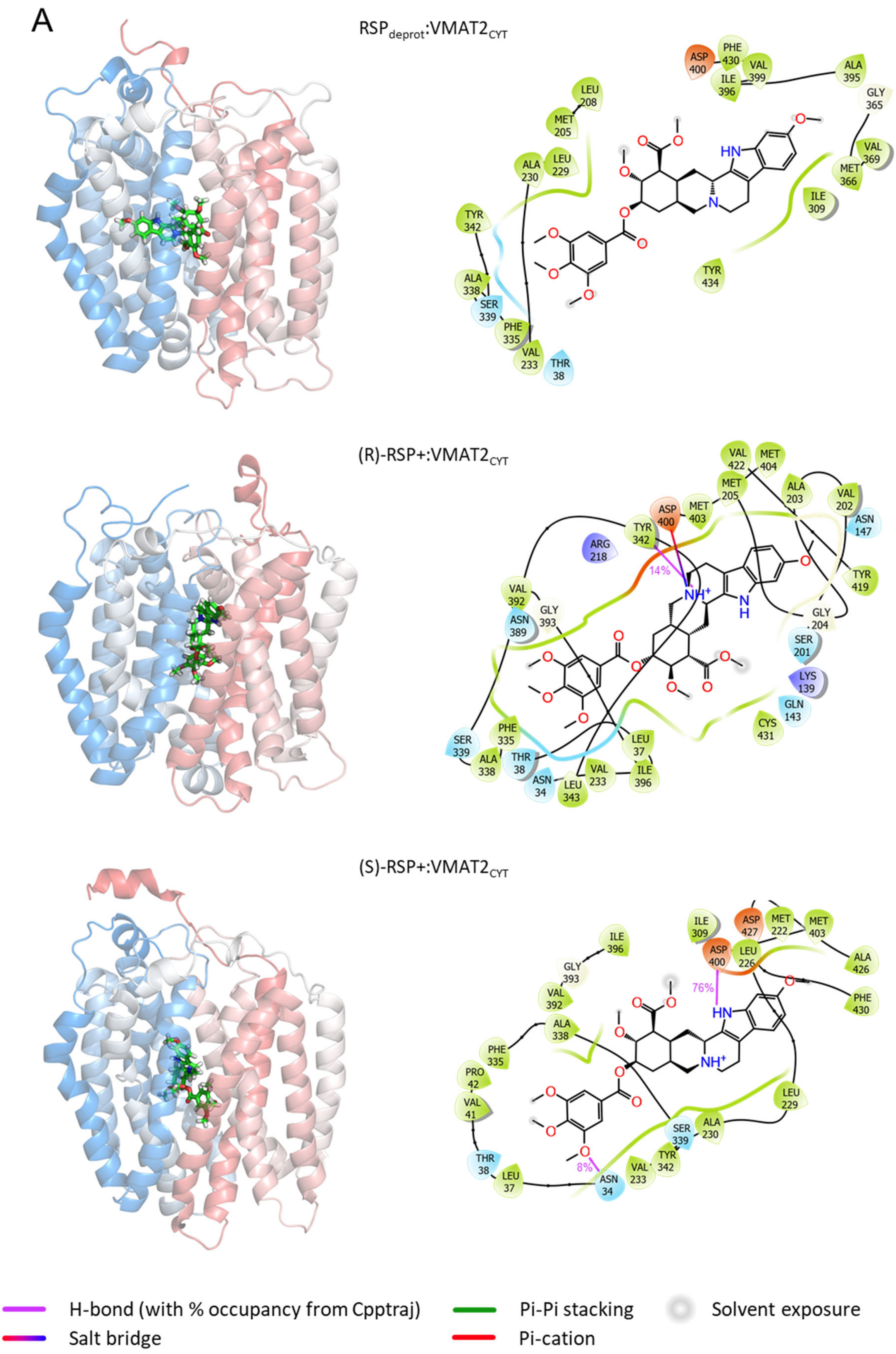
A
RSPdeprot:VMAT2CYT
ASP 400
PHE 430
VAL 399
ILE 396
ALA 395
GLY 365
LEU 208
MET 205
ALA 230
LEU 229
VAL 369
MET 366
ILE 309
TYR 342
TYR 434
ALA 338
SER 339
PHE 335
VAL 233
THR 38
(R)-RSP+:VMAT2CYT
VAL 422
MET 404
MET 205
ALA 203
VAL 202
ASN 147
ASP 400
MET 403
TYR 342
ARG 218
14%
TYR 419
VAL 392
GLY 393
ASN 389
GLY 204
SER 201
LYS 139
GLN 143
CYS 431
SER 339
PHE 335
ALA 338
THR 38
ASN 34
LEU 343
VAL 233
LEU 37
ILE 396
(S)-RSP+:VMAT2CYT
ILE 309
ASP 427
MET 222
MET 403
ILE 396
ASP 400
LEU 226
ALA 426
GLY 393
76%
PHE 430
VAL 392
ALA 338
PHE 335
PRO 42
VAL 41
LEU 229
SER 339
ALA 230
TYR 342
THR 38
8%
LEU 37
ASN 34
VAL 233
H-bond (with % occupancy from Cpptraj)
Salt bridge
Pi-Pi stacking
Pi-cation
Solvent exposure

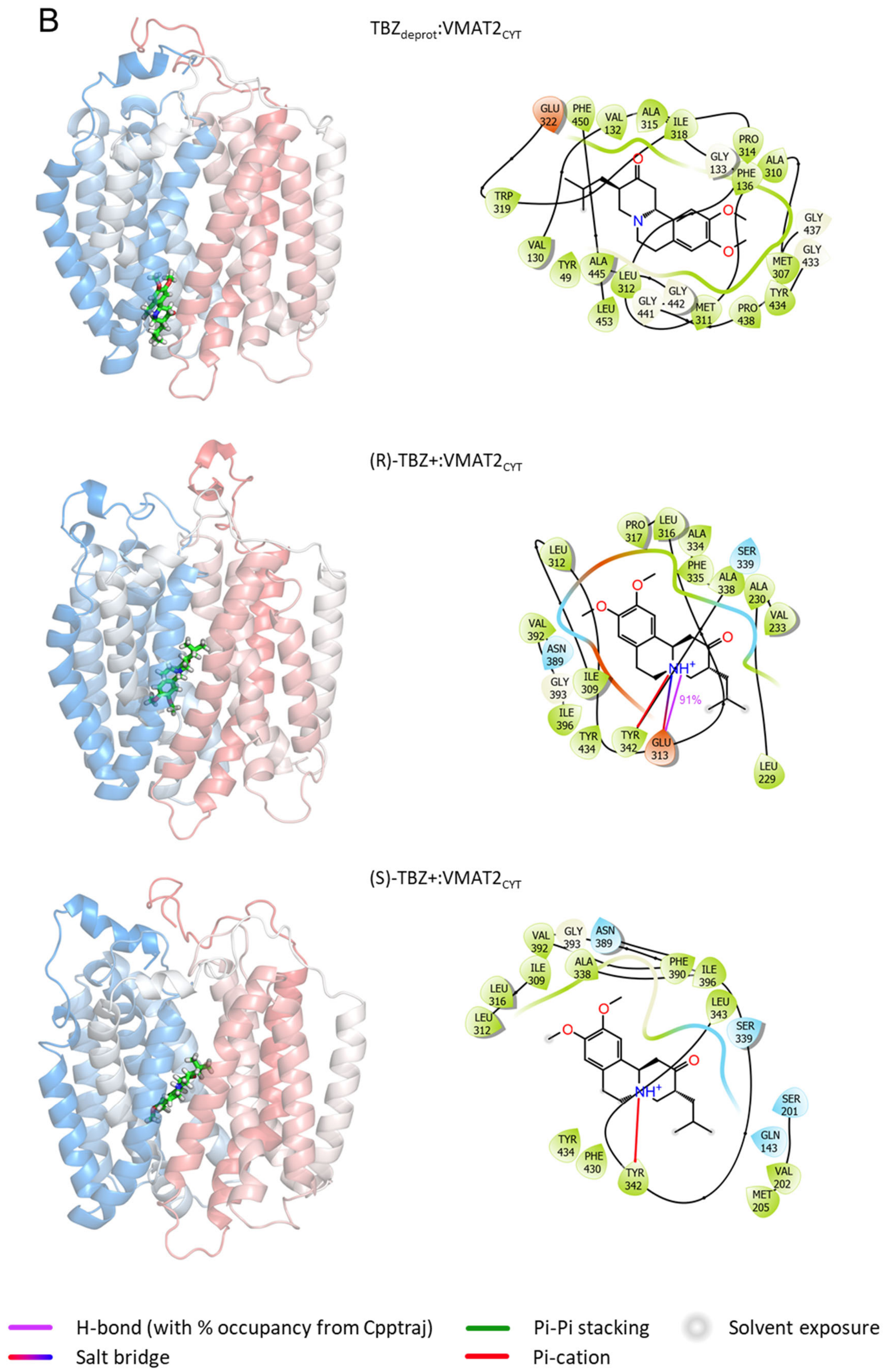

B
TBZ$_{deprot}$:VMAT2$_{CYT}$
GLU 322
PHE 450
VAL 132
ALA 315
ILE 318
PRO 314
GLY 133
ALA 310
PHE 136
TRP 319
GLY 437
GLY 433
VAL 130
TYR 49
ALA 445
LEU 312
MET 307
TYR 434
GLY 442
GLY 441
MET 311
PRO 438
LEU 453
(R)-TBZ+:VMAT2$_{CYT}$
PRO 317
LEU 316
ALA 334
SER 339
LEU 312
PHE 335
ALA 338
ALA 230
VAL 233
VAL 392
ASN 389
ILE 309
GLY 393
91%
ILE 396
TYR 434
TYR 342
GLU 313
LEU 229
(S)-TBZ+:VMAT2$_{CYT}$
VAL 392
GLY 393
ASN 389
ILE 309
ALA 338
PHE 390
ILE 396
LEU 316
LEU 343
LEU 312
SER 339
SER 201
GLN 143
TYR 434
PHE 430
TYR 342
VAL 202
MET 205
H-bond (with % occupancy from Cpptraj)
Salt bridge
Pi-Pi stacking
Pi-cation
Solvent exposure

C

(R)-SMT+:VMAT2$_{CYT}$

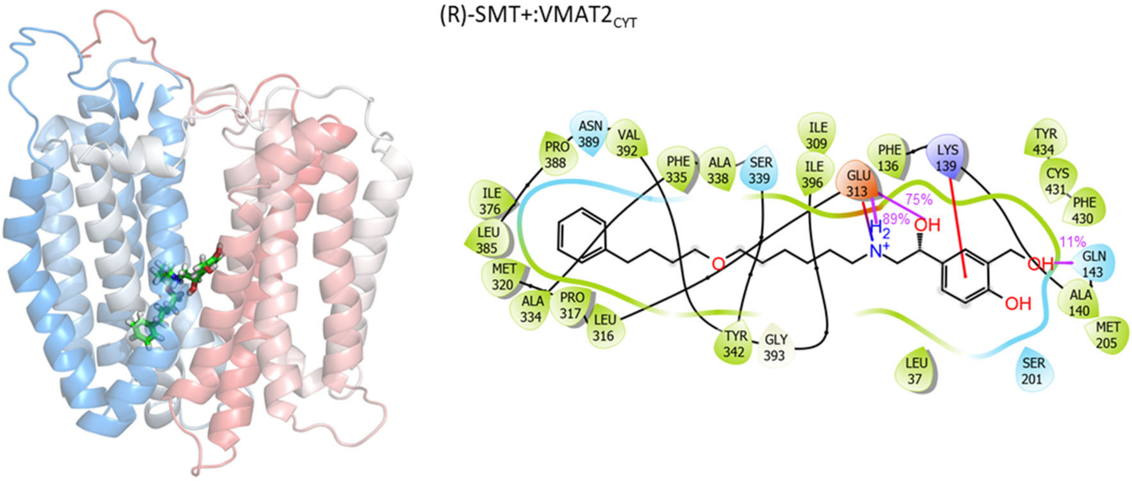

(S)-SMT+:VMAT2$_{CYT}$

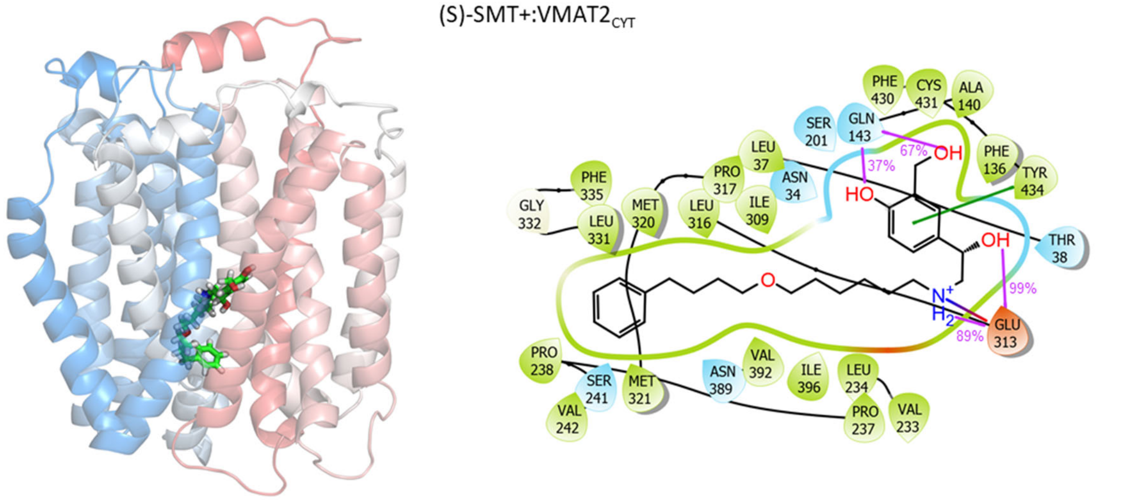

H-bond (with % occupancy from Cpptraj)

Salt bridge

Pi-Pi stacking

Pi-cation

Solvent exposure

D

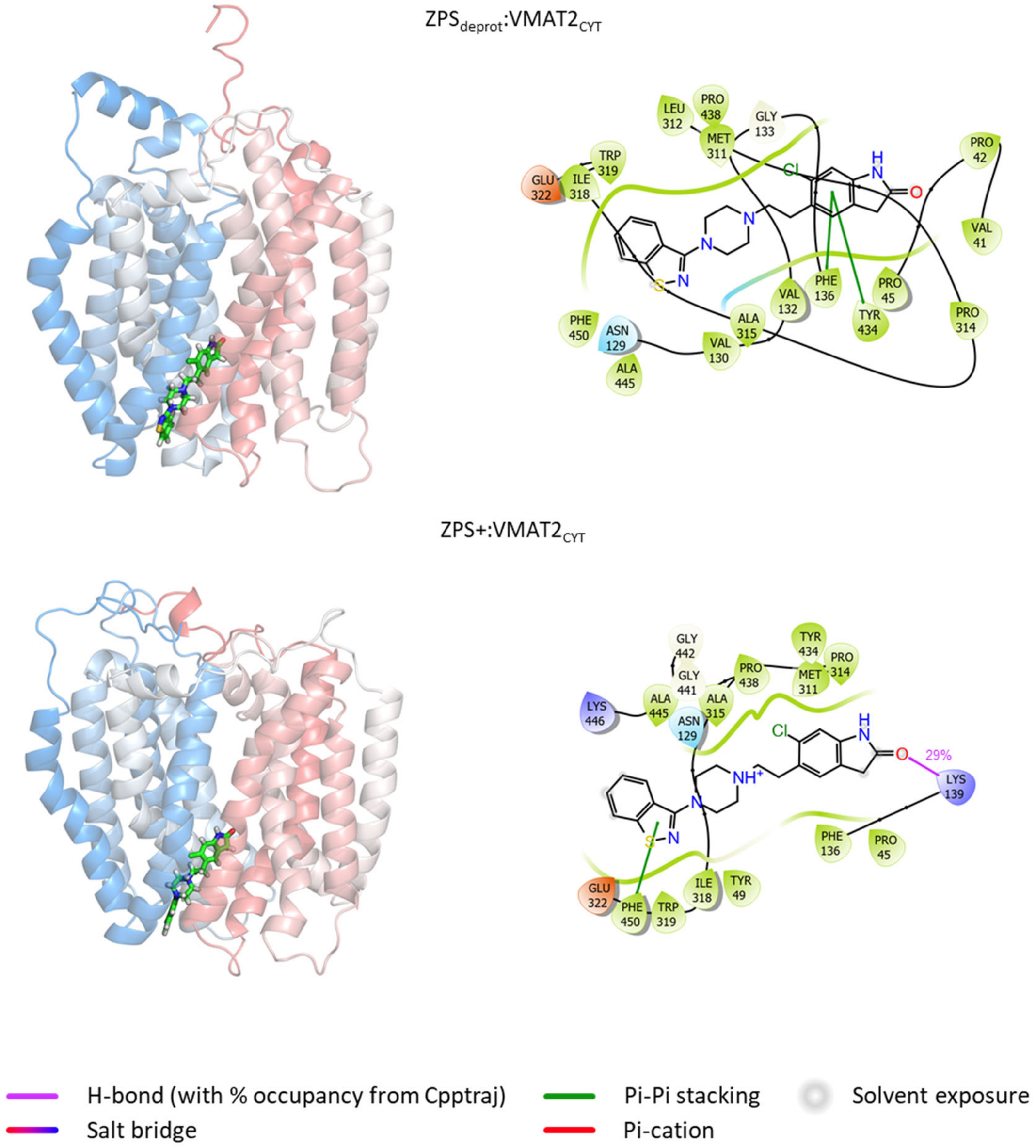

ZPS$_{deprot}$:VMAT2$_{CYT}$
LEU 312
PRO 438
GLY 133
MET 311
TRP 319
GLU 322
ILE 318
PRO 42
VAL 41
PHE 136
PRO 45
VAL 132
TYR 434
PRO 314
PHE 450
ASN 129
ALA 315
VAL 130
ALA 445
ZPS+:VMAT2$_{CYT}$
GLY 442
GLY 441
TYR 434
PRO 314
PRO 438
MET 311
LYS 446
ALA 445
ALA 315
ASN 129
29%
LYS 139
PHE 136
PRO 45
GLU 322
PHE 450
TRP 319
ILE 318
TYR 49
H-bond (with % occupancy from Cpptraj)
Salt bridge
Pi-Pi stacking
Pi-cation
Solvent exposure

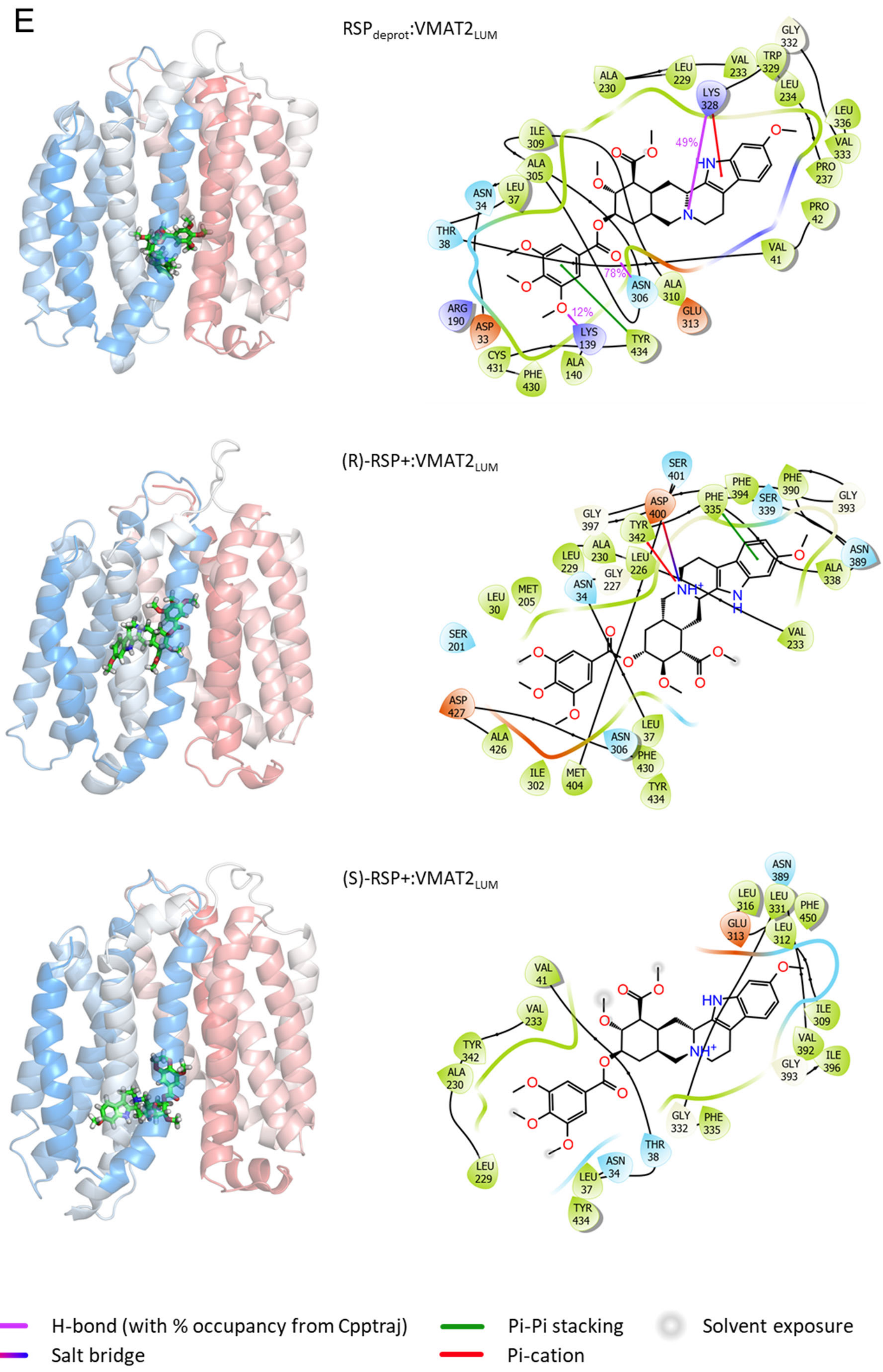
E
RSP$_{deprot}$:VMAT2$_{LUM}$
GLY 332
ALA 230
LEU 229
VAL 233
TRP 329
LEU 234
LYS 328
LEU 336
VAL 333
ILE 309
49%
ALA 305
PRO 237
ASN 34
LEU 37
PRO 42
THR 38
VAL 41
78%
ASN 306
ALA 310
GLU 313
ARG 190
ASP 33
12%
LYS 139
TYR 434
CYS 431
PHE 430
ALA 140
(R)-RSP+:VMAT2$_{LUM}$
SER 401
PHE 394
PHE 390
ASP 400
PHE 335
SER 339
GLY 393
GLY 397
TYR 342
ALA 230
LEU 229
LEU 226
ASN 389
ALA 338
GLY 227
ASN 34
LEU 30
MET 205
SER 201
VAL 233
ASP 427
ALA 426
ASN 306
LEU 37
PHE 430
ILE 302
MET 404
TYR 434
(S)-RSP+:VMAT2$_{LUM}$
ASN 389
LEU 316
LEU 331
PHE 450
GLU 313
LEU 312
VAL 41
ILE 309
VAL 233
VAL 392
ILE 396
TYR 342
GLY 393
ALA 230
GLY 332
PHE 335
THR 38
LEU 229
ASN 34
LEU 37
TYR 434
H-bond (with % occupancy from Cpptraj)
Salt bridge
Pi-Pi stacking
Pi-cation
Solvent exposure

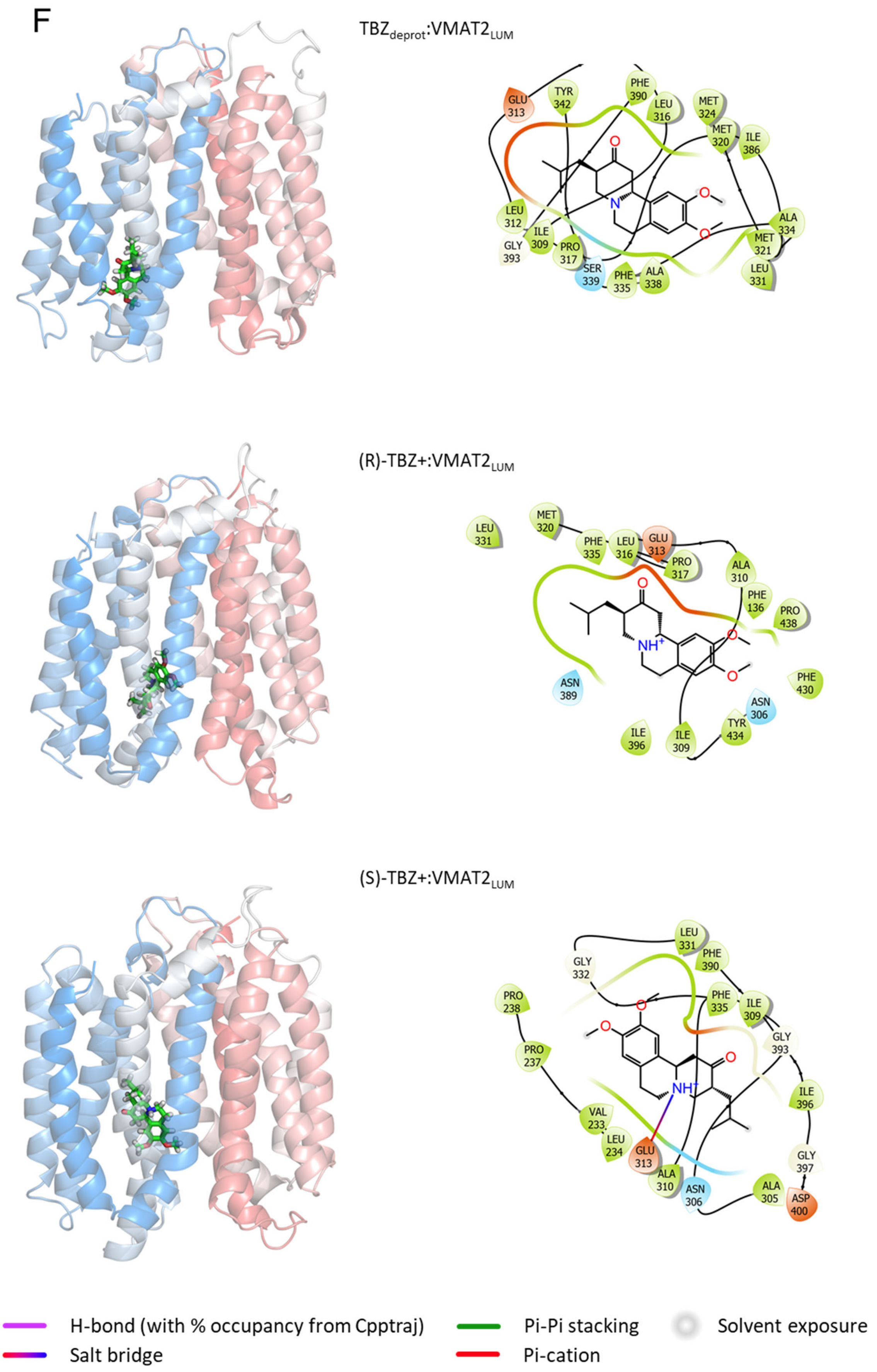
F
TBZdeprot:VMAT2LUM
GLU 313
TYR 342
PHE 390
LEU 316
MET 324
MET 320
ILE 386
LEU 312
ILE 309
PRO 317
GLY 393
SER 339
PHE 335
ALA 338
MET 321
ALA 334
LEU 331
(R)-TBZ+:VMAT2LUM
LEU 331
MET 320
PHE 335
LEU 316
GLU 313
PRO 317
ALA 310
PHE 136
PRO 438
NH+
ASN 389
PHE 430
ASN 306
TYR 434
ILE 396
ILE 309
(S)-TBZ+:VMAT2LUM
LEU 331
PHE 390
GLY 332
PRO 238
PHE 335
ILE 309
GLY 393
PRO 237
NH+
ILE 396
VAL 233
LEU 234
GLU 313
ALA 310
ASN 306
ALA 305
GLY 397
ASP 400
H-bond (with % occupancy from Cpptraj)
Salt bridge
Pi-Pi stacking
Pi-cation
Solvent exposure

G

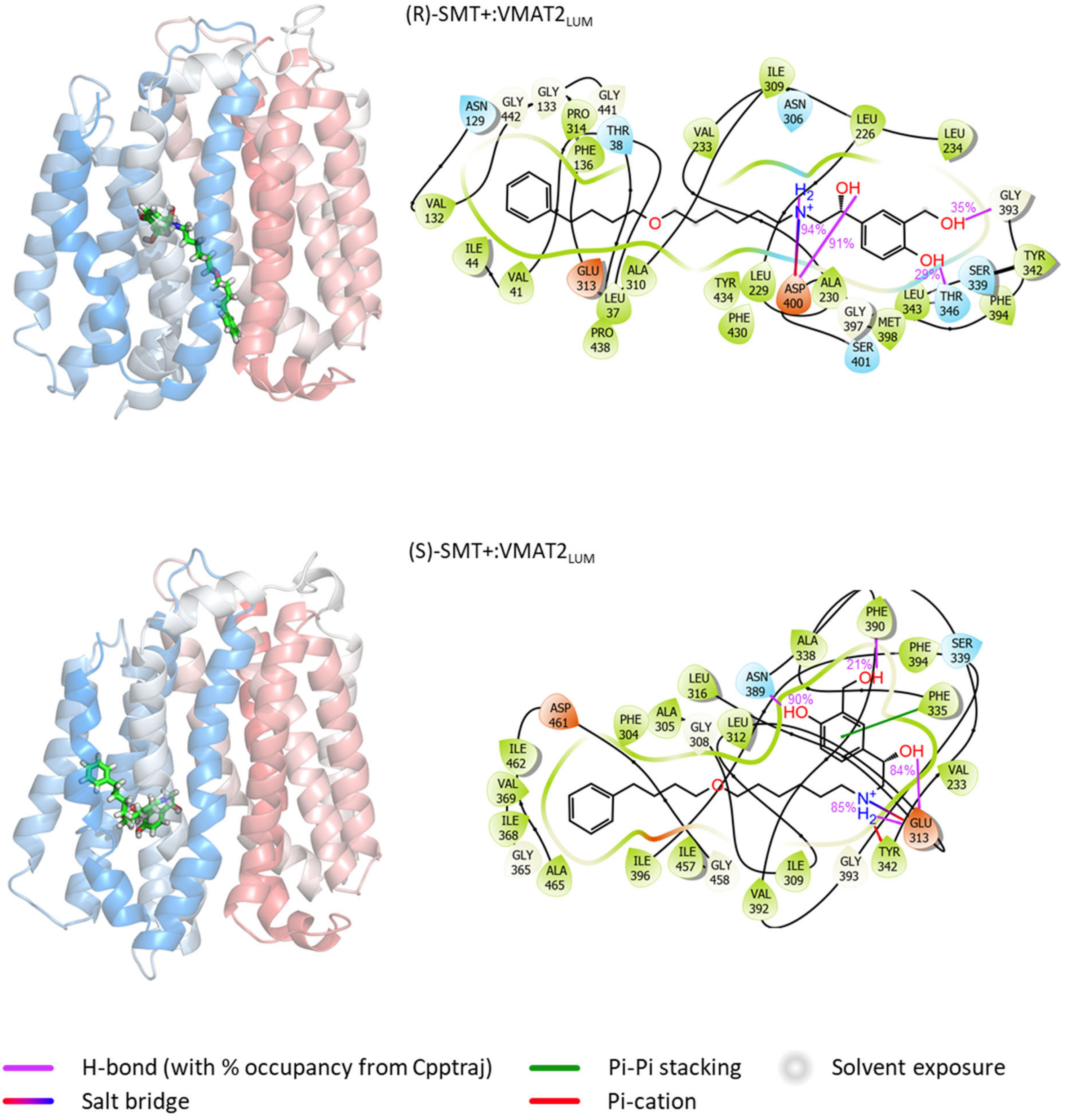

(R)-SMT+:VMAT2LUM
ASN 129
GLY 442
GLY 133
GLY 441
PRO 314
THR 38
PHE 136
ILE 309
ASN 306
VAL 233
LEU 226
LEU 234
GLY 393
VAL 132
ILE 44
VAL 41
GLU 313
ALA 310
LEU 37
PRO 438
TYR 434
PHE 430
LEU 229
ASP 400
ALA 230
GLY 397
SER 401
MET 398
LEU 343
THR 346
SER 339
PHE 394
TYR 342
O
H2
N+
OH
OH
OH
94%
91%
35%
29%
(S)-SMT+:VMAT2LUM
PHE 390
ALA 338
PHE 394
SER 339
LEU 316
ASN 389
PHE 335
ASP 461
PHE 304
ALA 305
GLY 308
LEU 312
ILE 462
VAL 369
ILE 368
GLY 365
ALA 465
ILE 396
ILE 457
GLY 458
VAL 392
ILE 309
GLY 393
TYR 342
GLU 313
VAL 233
O
N+
H2
HO
OH
OH
21%
90%
84%
85%
H-bond (with % occupancy from Cpptraj)
Salt bridge
Pi-Pi stacking
Pi-cation
Solvent exposure

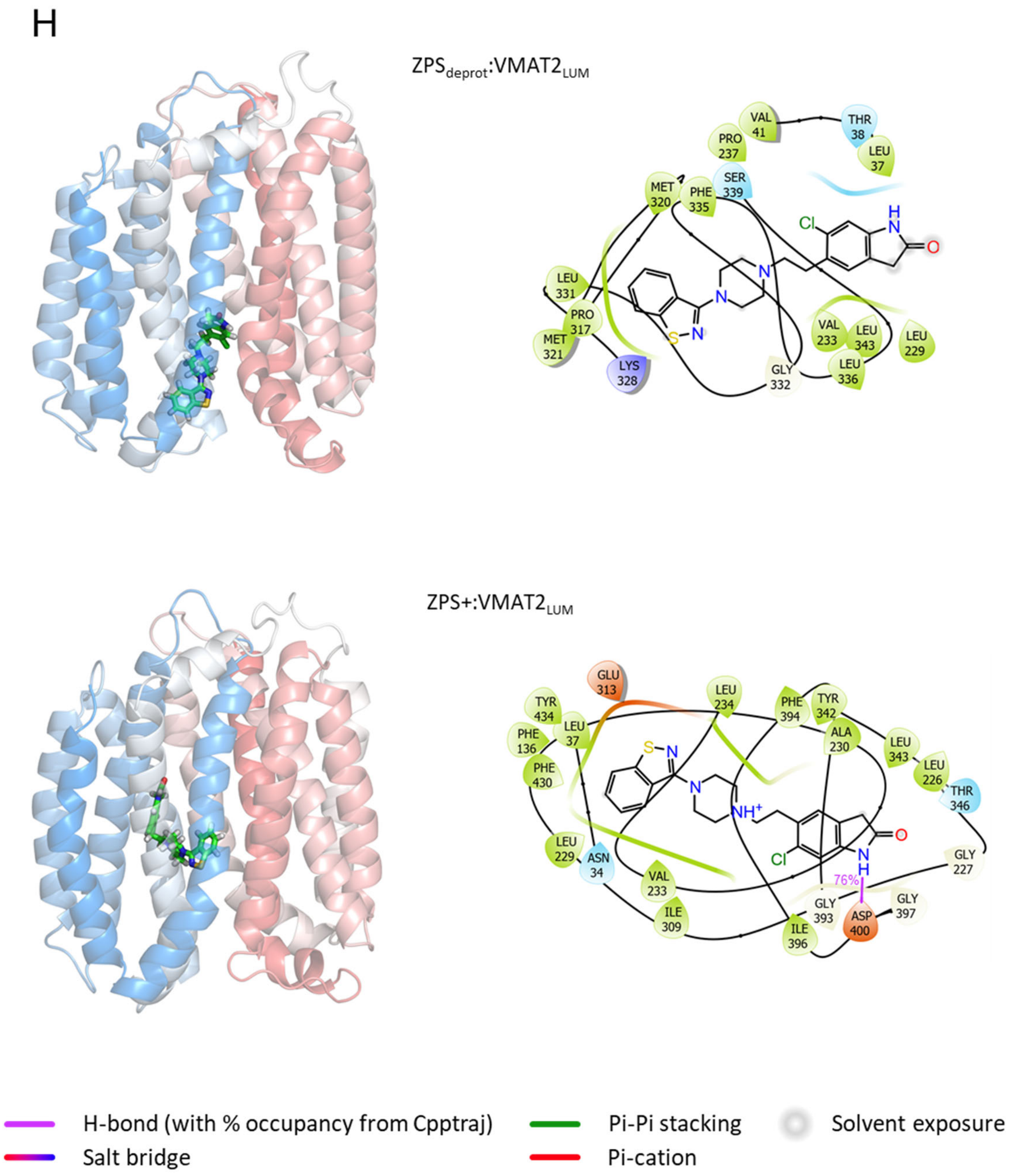

Supplementary Figure 3. Ligand:VMAT2 binding modes from simulations. Panels A-H provide the representative binding modes from the stable, last 200 ns portions of the ligand:VMAT2 simulations, both as 3D representations of the ligands in complex with the entire protein (left, ligand shown as sticks, VMAT2 shown as cartoon colored by sequence from the N-terminal in red to the C-terminal in blue) and as 2D interaction diagrams (right) displaying the binding pockets defined by the VMAT2 residues within 4 Å of the ligand. The representative structure in each case was extracted as the centroid of the largest cluster following a clustering in CPPTRAJ based on the ligand position in the already RMSD-fitted protein. The 2D ligand interaction diagrams were generated using Schrödinger Maestro (Release 2019-3: Maestro, Schrödinger, LLC, New York, NY, 2019), and hydrogen bond occupancies calculated from the simulations using CPPTRAJ were added to the diagrams.

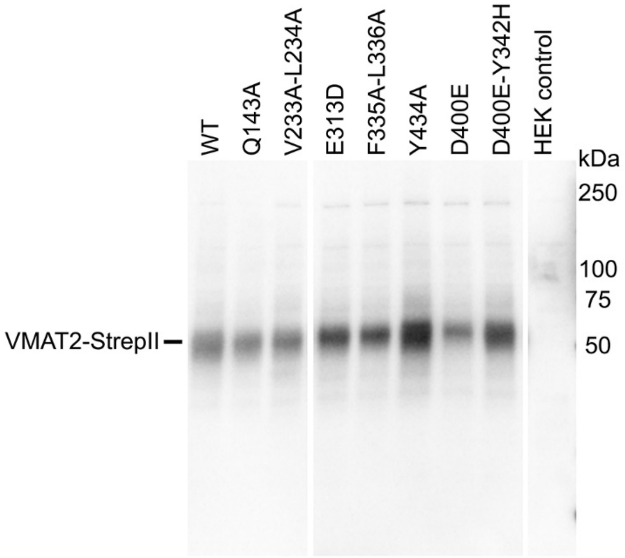

Supplementary Figure 4. Representative western blot used for quantification and normalization of data presented in Figure 6. The amount of rat VMAT2-Strep-tagII present in each sample was quantified by SDS-PAGE and western blotting using a StrepMAB-Classic HRP conjugate (IBA LifeSciences GmbH). Western blots were imaged using a Chemidoc XRS gel imaging system (Bio-Rad), and the indicated VMAT2-StrepII bands were quantified using the Image Lab software from Bio-Rad.

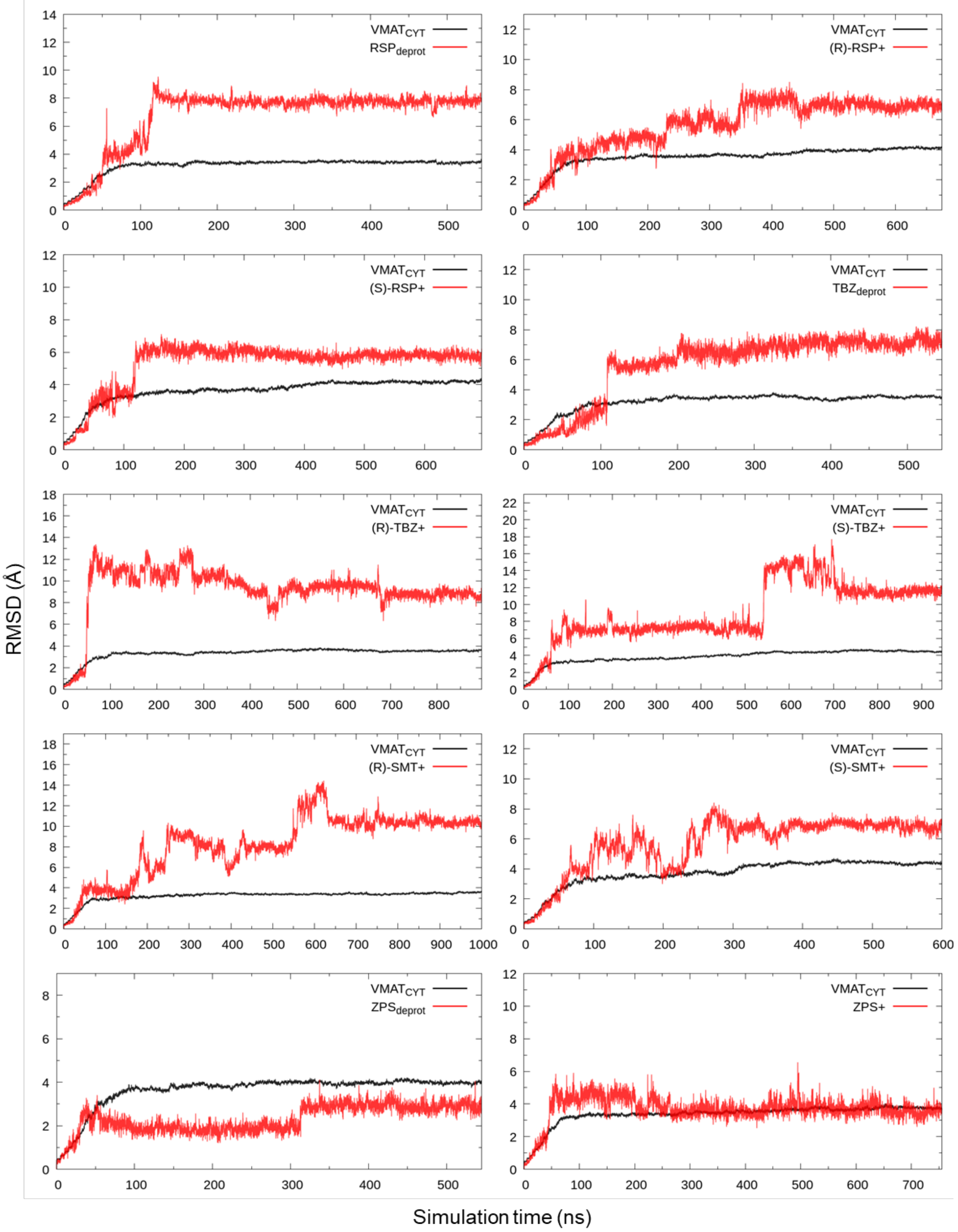

Supplementary Figure 5. RMSD from ligand:VMAT2$_{CYT}$ simulations. RMSD fitting to the VMAT2 transmembrane Cα atoms (black line) was followed by "nofit" RMSD of the ligand heavy atoms (red line) as an expression of the positional stability of the ligand relative to the fitted protein. The last 200 ns of each simulation was used for analysis.

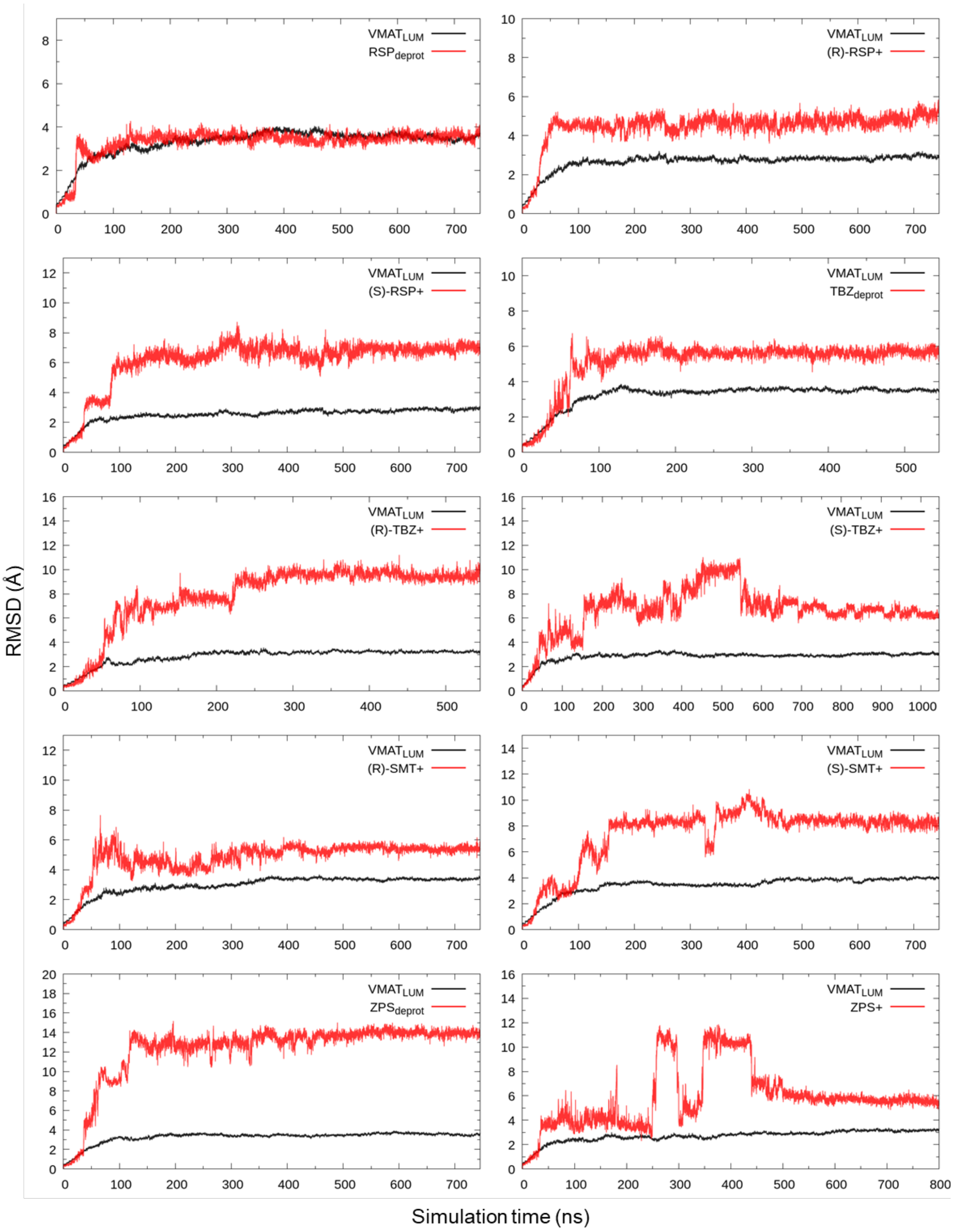

Supplementary Figure 6. RMSD from ligand:VMAT2$_{LUM}$ simulations. RMSD fitting to the VMAT2 transmembrane Cα atoms (black line) was followed by "nofit" RMSD of the ligand heavy atoms (red line) as an expression of the positional stability of the ligand relative to the fitted protein. The last 200 ns of each simulation was used for analysis.

References

1. Ugolev, Y., et al., *Identification of conformationally sensitive residues essential for inhibition of vesicular monoamine transport by the noncompetitive inhibitor tetrabenazine.* J Biol Chem, 2013. **288**(45): p. 32160-71.
2. Gros, Y. and S. Schuldiner, *Directed evolution reveals hidden properties of VMAT, a neurotransmitter transporter.* J Biol Chem, 2010. **285**(7): p. 5076-84.
3. Yaffe, D., et al., *Identification of molecular hinge points mediating alternating access in the vesicular monoamine transporter VMAT2.* Proc Natl Acad Sci U S A, 2013. **110**(15): p. E1332-41.
4. Yaffe, D., et al., *Emulating proton-induced conformational changes in the vesicular monoamine transporter VMAT2 by mutagenesis.* Proc Natl Acad Sci U S A, 2016. **113**(47): p. E7390-E7398.
5. Finn, J.P., 3rd and R.H. Edwards, *Multiple residues contribute independently to differences in ligand recognition between vesicular monoamine transporters 1 and 2.* J Biol Chem, 1998. **273**(7): p. 3943-7.
6. Larkin, M.A., et al., *Clustal W and Clustal X version 2.0.* Bioinformatics, 2007. **23**(21): p. 2947-8.
7. Robert, X. and P. Gouet, *Deciphering key features in protein structures with the new ENDscript server.* Nucleic Acids Res, 2014. **42**(Web Server issue): p. W320-4.
