# Supplementary material for: Inhibition of VMAT2 by β2-adrenergic agonists, antagonists, and the atypical antipsychotic ziprasidone
Source: Commun Biol. 2022 Nov 23;5:1283. doi: 10.1038/s42003-022-04121-1 (PMC9684503; doi:10.1038/s42003-022-04121-1)
Supplement: Supplementary file 2 — Description of Additional Supplementary Files [file 42003_2022_4121_MOESM2_ESM.docx]

**Description of Additional Supplementary Files**

**File name:** Supplementary Data 1

**Description:** Supplementary Data 1 contains source data for all charts and figures in the manuscript.
